# Anti-inflammatory therapies to prevent cardiovascular events: systematic review and network meta-analysis of randomised controlled trials

**DOI:** 10.3389/fcvm.2026.1717817

**Published:** 2026-03-11

**Authors:** Kevin E. Boczar, Alexander L. Pearson, Ramtin Hakimjavadi, Sheojung Shin, Saba Shahab, Aishwarya Geejo, Sarah M. Visintini, Christopher A. Fehlmann, Kathryn A. Bezzina, Rob S. B. Beanlands, George A. Wells

**Affiliations:** 1 Department of Cardiology, University of Ottawa Heart Institute, Ottawa, ON, Canada; 2 School of Epidemiology and Public Health, University of Ottawa, Ottawa, ON, Canada; 3 Department of Medicine, University of Pennsylvania, Philadelphia, PA, United States; 4 Care of the Elderly Program, Élisabeth Bruyère Hospital, Ottawa, ON, Canada; 5 Berkman Library, University of Ottawa Heart Institute, Ottawa, ON, Canada; 6 Ottawa Hospital Research Institute, Ottawa, ON, Canada; 7 Division of Emergency Medicine, Geneva University Hospitals, Geneva, Switzerland; 8 Research Methods Centre, University of Ottawa Heart Institute, Ottawa, ON, Canada

**Keywords:** acute coronary syndrome, anti-inflammatory, coronary artery disease, network meta-analyses, therapeutics

## Abstract

**Background:**

Anti-inflammatory therapies have been increasingly investigated for the reduction of cardiovascular (CV) events. The objective of this paper was to summarize and compare the relative effectiveness of anti-inflammatory medications for the reduction of CV events in patients with known coronary artery disease (CAD), either acute coronary syndromes (ACS) or stable CAD.

**Methods:**

Systematic review and network meta-analysis of randomised controlled trials (RCTs) that included at least one anti-inflammatory treatment and involved patients with CAD. Databases searched: Medline, Embase, Cochrane Central Register of Controlled Trials, clinical trial registry websites, Europe PMC, and conference abstracts. Bayesian network meta-analysis was performed to calculate risk estimates using fixed-effects analyses in patients with ACS and stable CAD. Risk of bias assessments were performed using the Cochrane Risk of Bias 2 (RoB2) tool.

**Results:**

17,021 studies were screened; 41 met inclusion criteria. 29,487 patients were included in the ACS network and 41,791 in the stable CAD network. In the ACS network analysis, both non-steroidal anti-inflammatory drugs [OR: 0.30, 95% Credible Limits (CrI): 0.11–0.74] and colchicine (OR: 0.77, CrI: 0.62–0.95) were associated with a significant reduction in major adverse cardiac events (MACE) compared to control. In the stable CAD analysis, both corticosteroids (OR: 0.44, 95% CrI: 0.26–0.72) and colchicine (OR: 0.65, CrI: 0.54–0.77) were associated with a significant reduction in MACE compared to control.

**Conclusions:**

In patients with ACS, colchicine was associated with a reduction in MACE, while observed associations for NSAIDs were derived from sparse and predominantly indirect evidence. In patients with stable CAD, colchicine and corticosteroids were associated with a reduction in MACE, although these findings were informed largely by indirect comparisons.

**Systematic Review Registration:**

Identifier CRD42022303289.

## Introduction

CAD is a leading cause of morbidity and mortality worldwide. Great strides have been made in the secondary prevention of CAD, with the goal of mitigating its resultant complications such as myocardial infarction and left ventricular dysfunction. While therapies such as antiplatelets, angiotensin-converting enzyme inhibitors, and medications targeting low-density lipoprotein are well-established parts of routine clinical care, more attention has recently been given to anti-inflammatory therapies (1, 2).

Inflammation has long been recognized to play an integral role in the pathogenesis of atherosclerotic disease. However, it has only been in recent years that inflammation has been regarded as a therapeutic target (3). Recent guidelines (e.g., Canadian, European, and South American) (4–6) now recommend using low-dose colchicine for secondary prevention of cardiovascular events in patients with CAD. Furthermore, numerous trials have shown varying levels of CV benefit using therapies to target several aspects of the inflammatory cascade.

While some anti-inflammatory therapies have demonstrated effectiveness relative to placebo in randomized controlled trials, the relative efficacy of these therapies compared to each other is unclear. Therefore, the objective of this study was to compare anti-inflammatory agents for the secondary prevention of CV disease in patients with known CAD, both in the ACS setting and the stable CAD setting, in terms of their relative effectiveness and side effects.

## Methods

This review is reported according to PRISMA-NMA guidelines (7).

A peer-reviewed search was conducted on January 15, 2025 in MEDLINE, Embase, and the Cochrane Central Register of Controlled Trials via Ovid (Supplementary Tables S1-S3) (8). No limits to language or publication date were applied. However, RCT search filters were applied in Medline and Embase (9). Regular alerts were also set up for Medline. The protocol was published and registered in the PROSPERO International Prospective Register of Systematic Reviews (CRD42022303289) (10).

Grey literature searching included searches of clinical trial registries, a search of Europe PMC for pre-prints, and hand-searching of select conferences for any abstracts not already indexed in Embase (Supplementary Tables S4-S7).

Search results were exported to Covidence (Melbourne, Australia) and duplicates were eliminated using the platform's duplicate identification feature.

The population that we studied were individuals with coronary artery disease (both ACS and stable CAD). The following anti-inflammatory treatments were included: corticosteroids, methotrexate, tocilizumab, anakinra, canakinumab, pexelizumab, colchicine, NSAIDs, EA-230, LeukArrest, darapladib, varespladib, losmapimod, succinobucol, and inclacumab.

RCTs with either active- or placebo-control groups were included if they evaluated at least one anti-inflammatory drug under review, reported data for MACE, or any of its subcomponents, and enrolled patients with known coronary artery disease. Of note, definitions of MACE varied across trials. Most included cardiovascular death and myocardial infarction, with variable inclusion of stroke, urgent revascularization, or hospitalization for ischemia. For this analysis, we accepted trial-defined MACE as reported, consistent with prior cardiovascular meta-analyses. Studies with non-ischemic or idiosyncratic composite definitions were excluded. We included both published and yet-to-be-published research (e.g., posters). Two reviewers independently reviewed the abstracts and full texts of retrieved articles to ensure they met inclusion criteria. For the included articles, data was independently extracted by two reviewers in parallel. Disagreements were resolved by a third investigator. Risk of bias assessment of RCTs was performed independently by two reviewers using the Cochrane Risk of Bias 2 (RoB2) tool (11).

Bayesian network meta-analysis was performed utilizing WinBUGS software (MRC Biostatistics Unit, Cambridge UK). We used a binomial likelihood model as it accounts for multi-arm trials within the evidence network. Trials with zero events in one study arm were retained in the network, as exclusion of such trials can bias effect estimates in sparse-event settings. A continuity correction of 0.5 was applied only when required to permit model estimation in studies with a single zero-event arm. No trials with zero events in both arms were included. Multi-arm trials were incorporated using methods that appropriately account for correlation between comparisons within the same study. In the Bayesian framework, all treatment arms were modeled simultaneously within a single likelihood, preserving randomization and avoiding double-counting of shared control groups. This approach yields valid variance estimation and is standard in network meta-analyses with multi-arm designs. A fixed effects network meta-analysis was utilized, as most of the evidence network consisted of single study connections. Given the substantial clinical heterogeneity across trials, including differences in study era, patient populations, interventions, and follow-up duration, a random-effects model would ordinarily be preferred. However, the evidence network was predominantly composed of single-study treatment comparisons, and random-effects models resulted in poor convergence and implausibly wide credible intervals, limiting interpretability. We therefore selected a fixed-effects approach to permit estimation across the network, while acknowledging the strong homogeneity assumption inherent to this model. Because of sparse network geometry and limited numbers of studies per comparison, network meta-regression was not feasible. We modelled odds ratio (OR) point estimates as well as 95% credible intervals using Markov Chain Monte Carlo methods. Hazard ratios were not used because time-to-event data were inconsistently reported across trials, particularly among older studies, and sufficient information was frequently unavailable to reconstruct survival estimates. To ensure consistency across the network and maximize study inclusion, odds ratios were therefore selected as the common effect measure. We also calculated the probability that each treatment was the most efficacious agent, the second best, the third best, and so on (12, 13). Non-informative (vague) priors were used for all treatment effect parameters. We assessed model fit based on the deviance information criterion (DIC) and compared the residual deviance to the number of unconstrained data points (14). Convergence was assessed using standard diagnostics, including visual inspection of trace plots and the Brooks–Gelman–Rubin statistic, with values close to 1.0 indicating satisfactory convergence (12).

Three independent Markov Chain Monte Carlo chains were fitted in WinBUGS for each analysis with at least 40,000 iterations following an initial burn-in of at least 40,000 iterations. A key assumption with network meta-analysis is that there is no conflict between direct and indirect evidence, termed consistency (15). We assessed consistency within the model by comparing deviance and DIC statistics in fitted consistency and inconsistency models (12, 15). We also plotted the posterior mean deviance of the individual study data points in the inconsistency model against the study data points from the consistency model to identify potential loops where inconsistency was present (12, 15).

To assess whether trials were comparable within a network meta-analysis we examined the baseline patient characteristics to ensure similarity. We found that baseline rates of MACE were higher in trials looking at populations involving ACS relative to stable CAD, so the decision was made to create these networks separately. The WinBUGS code and data needed to replicate our analyses are available online (14).

We conducted two sensitivity analyses. Network meta-analyses were repeated with (1) only studies that had at least 30 days of treatment and 30 days of follow-up included, and (2) only studies that had at least 30 days of treatment, 30 days of follow-up, and that were published 2010 or later. Both sensitivity analyses were performed for the ACS and stable CAD networks separately. These sensitivity analyses were prespecified to address concerns regarding evolving background therapies and procedural practices, including the transition from pre-statin and bare-metal stent eras to contemporary guideline-directed medical therapy.

We used the CINeMA approach to evaluate the credibility of our results (16).

## Results

**Figure 1:**
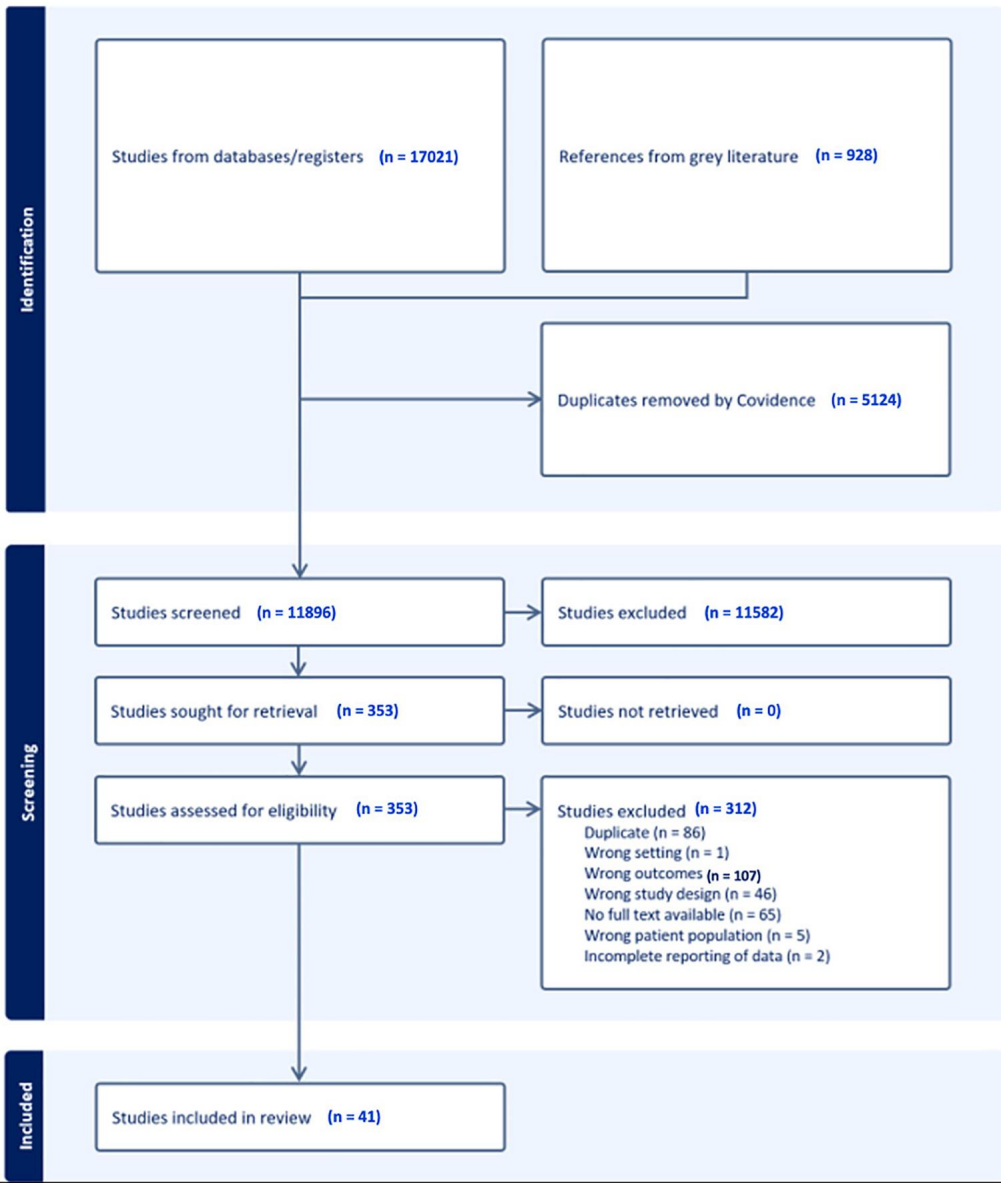
PRISMA diagram.

Our trial selection process is illustrated in a flow chart based on the Preferred Reporting Items for Systematic Reviews and Meta-Analyses (PRISMA) (7) statement (Figure 1).

A total of 17,021 studies were identified, and 41 RCTs met the eligibility criteria for inclusion (Supplementary Table S8). Within the ACS network for MACE, there were 21 studies included; trials evaluated the efficacy for the reduction of MACE in NSAIDs [1 study (17)], tocilizumab [2 studies (18, 19)], colchicine [10 studies (20–29)], anakinra [2 studies (30, 31)], darapladib [1 study (32)], LeukArrest [high and low dose; 1 study (33)], losmapimod [high and low dose; 2 studies (34, 35)], and varespladib [2 studies (36, 37)]. Most of the trials in the ACS network for MACE were published (20 studies) with 1 in poster format (26). Within the stable CAD network for MACE, there were 20 studies included; trials evaluated the efficacy for the reduction of MACE in corticosteroids [3 trials (38–40)], varespladib [2 studies (41, 42)], NSAIDs [3 studies (43–45)], inclacumab [1 study (46)], canakinumab [2 studies (47, 48)], colchicine [3 studies (22, 49, 50)], EA-230 [1 study (51)], darapladib [2 studies (52, 53)], methotrexate [1 study (54)], pexelizumab [1 study (55)], and succinobucol [1 study (56)]. All trials in the stable CAD network for MACE were in published format. Network, intervention, and direct comparison characteristics are presented in Tables 1 and 2 (network characteristics) and Supplementary Tables S9-S12 (intervention and direct comparison characteristics).

**Table 1:** Summary of acute coronary syndrome network characteristics.

Characteristic	Number
Number of Interventions	11
Number of Studies	21
Total Number of Patients in Network	29,487
Total Number of Events in Network	2,986
Total Possible Pairwise Comparisons	55
Total Number Pairwise Comparisons With Direct Data	12
Number of Two-arm Studies	16
Number of Multi-Arms Studies	2
Number of Studies With No Zero Events	18
Number of Studies With At Least One Zero Event	0
Number of Studies with All Zero Events	0

**Table 2:** Summary of stable coronary artery disease network characteristics.

Characteristic	Number
Number of Interventions	15
Number of Studies	20
Total Number of Patients in Network	41,791
Total Number of Events in Network	4,398
Total Possible Pairwise Comparisons	105
Total Number Pairwise Comparisons With Direct Data	18
Number of Two-arm Studies	18
Number of Multi-Arms Studies	2
Number of Studies With No Zero Events	20
Number of Studies With At Least One Zero Event	0
Number of Studies with All Zero Events	0

The NSAIDs and doses tested were: meloxicam 15 mg daily [for admission and 30 days following discharge (17)], celecoxib 400 mg before percutaneous angioplasty followed by 200 mg twice daily [for 3–6 months (42, 43)], or parecoxib/valdecoxib 40 mg every 12 h [for 14 days (41)]. The corticosteroids and doses used were: prednisone 1 mg/kg for 10–15 days followed by taper [0.5 mg/kg for 15–20 more days, then 0.25 mg/kg for 10–15 days (38, 40)], methylprednisolone 125 mg intramuscularly night before and morning of percutaneous angioplasty followed by prednisone 60 mg oral daily for one week (39). Colchicine was dosed at 0.5 mg daily [with a 2 mg loading dose (23) or without a loading dose (21, 25, 49, 50)], 1 mg daily (20, 24), or 1.8 mg once pre-procedure (22).

Baseline characteristics of studies are described in Supplementary Table S8. Mean age across the included trials varied from 57 to 73 years. All trials included patients of both sexes and most had a higher proportion of male participants. The studies were individually critically appraised. Overall, there was significant variation in study quality (Supplementary Table S13). The larger trials comprising the bulk of patients included in the systematic review were judged to be methodologically sound.

Bayesian network meta-analysis was performed to calculate risk estimates using fixed-effects analyses. There were 71,134 patients included in our analyses, with 29,487 patients included in the ACS network analysis and 41,791 patients included in the stable CAD network analysis. In the ACS analysis, colchicine [OR: 0.77, 95% Credible Limits (CrI): 0.62–0.95] and NSAID use (OR: 0.30, 95% CrI: 0.11–0.74) were associated with a significant reduction in MACE (Supplementary Table S14). In the stable CAD analysis, colchicine (OR: 0.65, CrI: 0.54–0.77) and corticosteroid use (OR: 0.44, 95% CrI): 0.26–0.72) were associated with a significant reduction in MACE. Most trials reported MACE as a primary outcome (Supplementary Table S15). Follow-up of trials reporting the primary outcome of MACE varied from 30 days to 4 years. Follow-up of trials reporting any outcome varied from 48 h to 4 years. Trials were published between the years of 1989 to 2021.

### Primary outcome: Major adverse cardiac events (MACE)

#### Acute coronary Syndromes (ACS) Network

**Figure 2:**
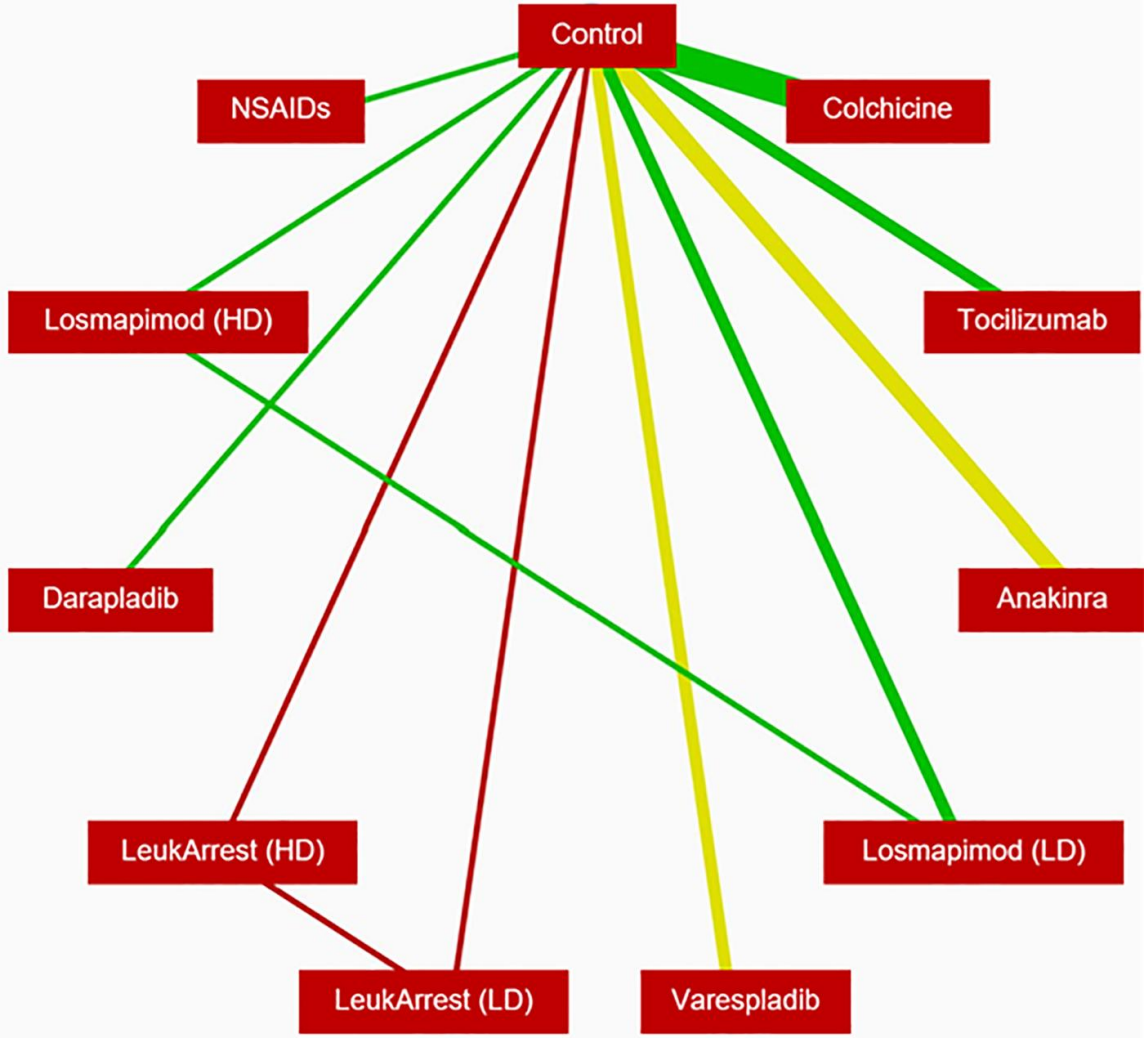
Evidence network for reduction of MACE in patients with ACS. The width of the lines is proportional to the number of randomised controlled trials comparing each pair of treatments, and the color of each line represents the average RoB of the trials comparing each pair of treatments (red is high RoB, yellow is moderate RoB, green is low RoB). MACE, major adverse cardiac events; ACS, acute coronary syndrome; RoB, risk of bias.

The evidence network (Figure 2) for the primary analysis of the ACS group was comprised of 21 RCTs representing 10 anti-inflammatory interventions in addition to placebo/control (*n* = 29,487). The posterior mean residual deviance (67.68) was somewhat close to the number of unconstrained data points (55), which is an indication of moderate model fit.

**Figure 3:**
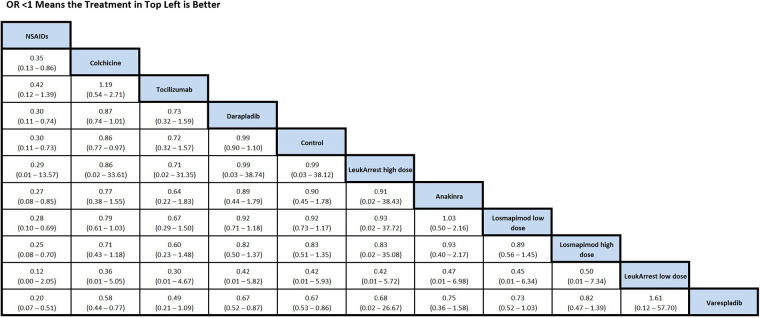
OR from network meta-analysis for MACE comparison in ACS network. Results between individual treatments should be interpreted with caution given the limitations associated with using a fixed-effects model. See Supplementary Table 9 for additional details. MACE=major adverse cardiac events, ACS, acute coronary syndrome; OR, odds ratio.

NSAIDs and colchicine were associated with a reduction in MACE relative to placebo/control (Figure 3). No differences in MACE were detected between standard therapy and each of the other anti-inflammatory therapies.

### Stable CAD network

**Figure 4:**
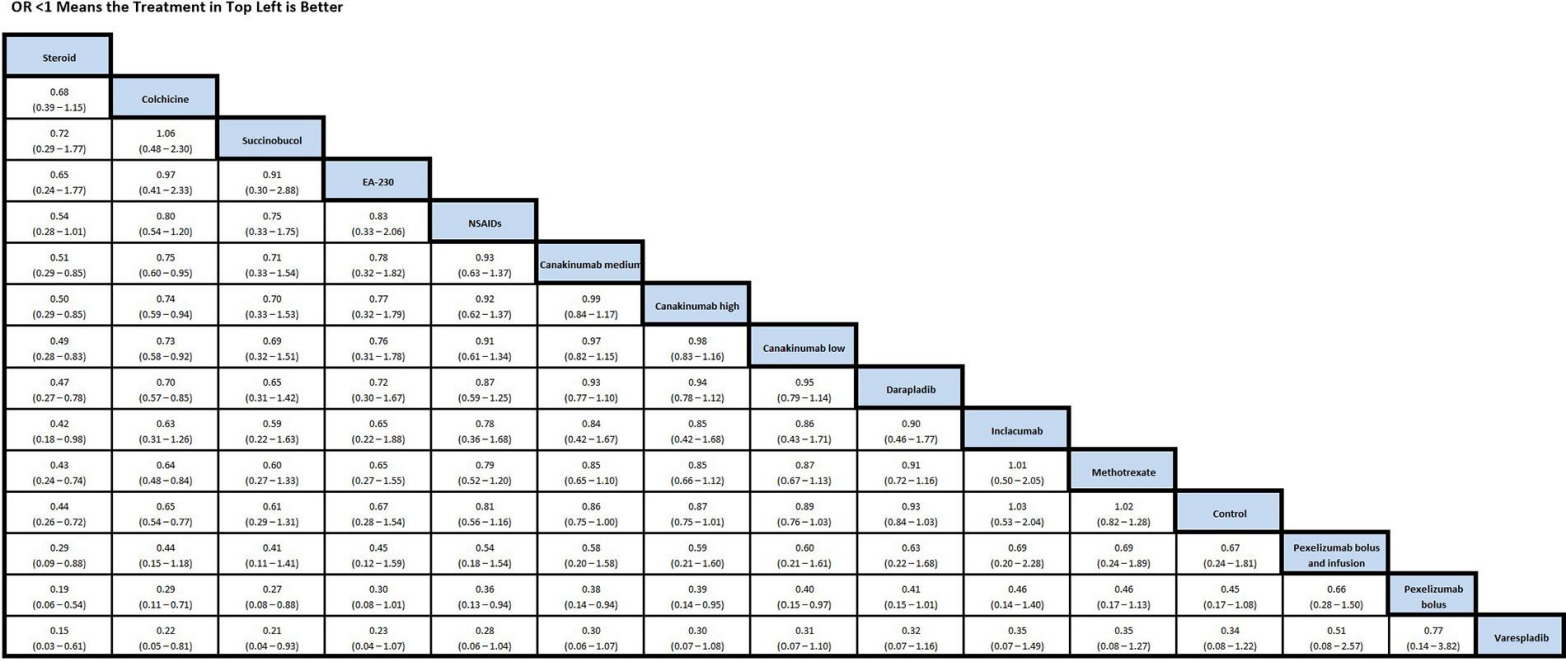
Evidence network for reduction of MACE in patients with stable CAD. The width of the lines is proportional to the number of randomised controlled trials comparing each pair of treatments, and the color of each line represents the average RoB of the trials comparing each pair of treatments (red is high RoB, yellow is moderate RoB, green is low RoB). MACE, major adverse cardiac events; CAD, coronary artery disease; RoB, risk of bias.

The evidence network (Figure 4) for the primary analysis for the stable coronary artery disease analysis was comprised of 20 RCTs representing 14 anti-inflammatory interventions in addition to placebo/control (*n* = 41,791). The posterior mean residual deviance (61.8) is less than the number of unconstrained data points (105), which is an indication of good model fit.

**Figure 5:**
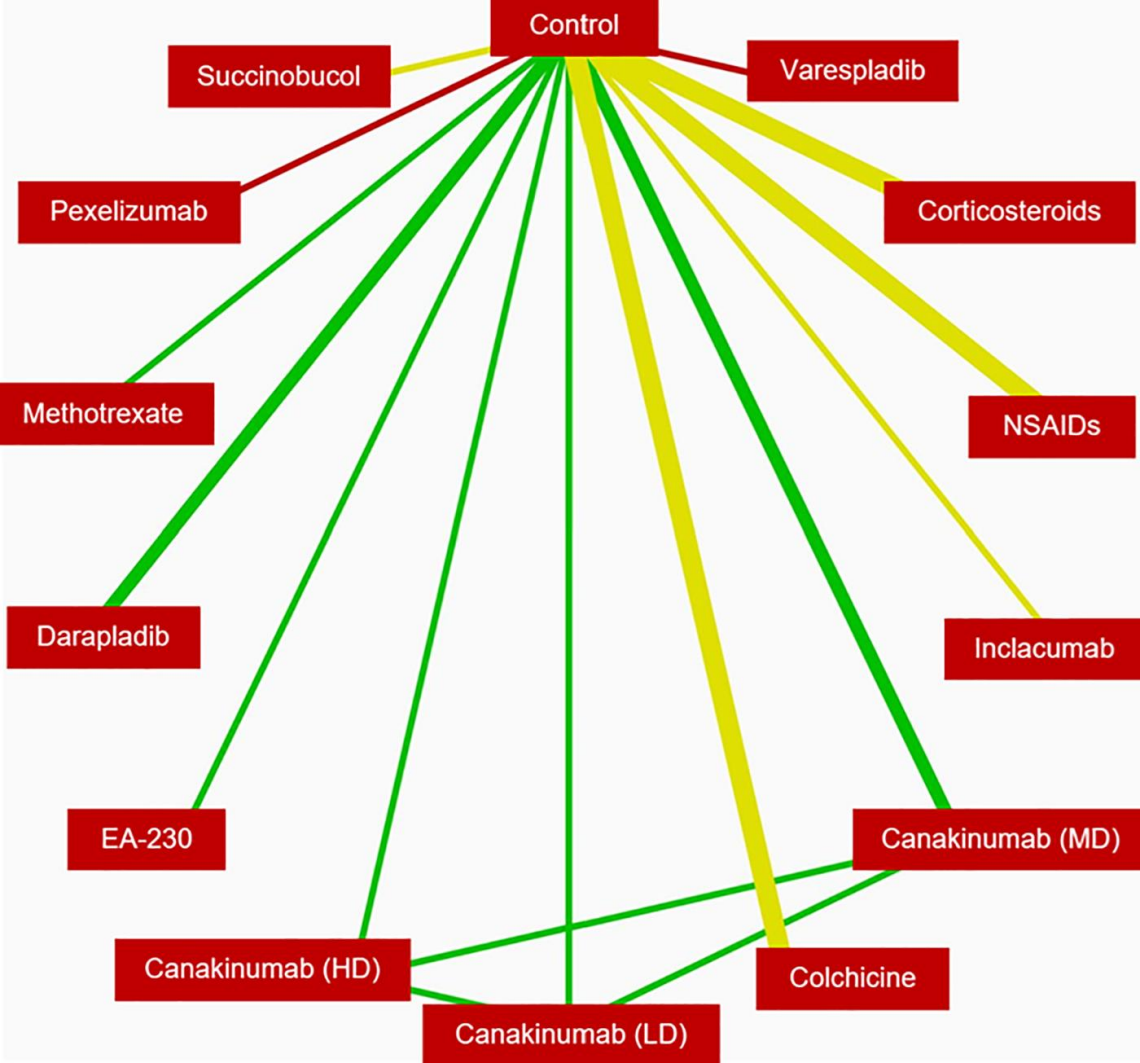
OR from network meta-analysis for MACE comparison in stable CAD network. Results between individual treatments should be interpreted with caution given the limitations associated with using a fixed-effects model. See Supplementary Table S10 for additional details. MACE, major adverse cardiac events; CAD, coronary artery disease; OR, odds ratio.

Both colchicine and corticosteroids were associated with a reduction in MACE relative to placebo/control (Figure 5). No differences in MACE were detected between standard therapy and each of the other anti-inflammatory therapies.

For both the ACS and stable CAD networks, estimates that were derived from the direct pairwise comparison of treatments were similar to those obtained in indirect comparisons. Additionally, comparison of the posterior mean deviance of the individual data points from fitted consistency and inconsistency models revealed no evidence of loops within the treatment network where inconsistency could be present in both the stable CAD and ACS networks, respectively.

### Confidence in the results

The CINeMA approach was used to assess the confidence in the results of this network meta-analysis (16). The full results are included in Supplementary Table S15. In both the ACS network and stable CAD network, the overall confidence in the results for each comparison made within the network was generally rated as either low or very low. This was largely secondary to the presence of significant imprecision and significant heterogeneity between included studies.

### Sensitivity analyses

#### ACS network

In the sensitivity analysis for the ACS network considering studies with ≥30 days of follow up and ≥30 days of treatment (12 studies, 35,286 patients; see Supplementary Table S16), only NSAID use was associated with a significant reduction in MACE (OR: 0.28, 95% CrI: 0.10–0.70). When further restricting to studies published 2010 or later (11 studies, 35,166 patients; see Supplementary Table S17), the one study evaluating NSAID use was excluded (Atman 2002), therefore no anti-inflammatory therapies were associated with a significant reduction in MACE.

### Stable CAD network

In the sensitivity analysis for the stable CAD network, there were 14 studies with ≥30 days of follow up and ≥30 days of treatment including 38,959 patients (Supplementary Table S18). Corticosteroids (OR: 0.37, 95% CrI: 0.21–0.65), colchicine (OR: 0.63, 95% CrI: 0.52–0.76), and NSAIDs (OR: 0.64, 95% CrI: 0.43–0.96), were all associated with a significant reduction in MACE. When restricting to studies published 2010 or later (11 studies with 38,088 patients; see Supplementary Table S19), corticosteroids (OR: 0.37, 95% CrI: 0.21–0.65), colchicine (OR: 0.63, 95% CrI: 0.52–0.76), and NSAID use (OR: 0.64, 95% CrI: 0.43–0.96) remained significantly associated with reduction in MACE.

### Secondary outcomes

Secondary outcomes included individual subcomponents of MACE and adverse effects of therapy (see Supplementary Materials). Overall, adverse event data were inconsistently reported and frequently underpowered, therefore quantitative analyses was not pursued. Instead, adverse events and harms were summarized systematically by drug class to contextualize potential net clinical benefit. A structured overview is provided in Table 3.

**Table 3:** Summary of adverse events by drug class across ACS and stable CAD networks.

Drug class	Network	Safety outcomes assessed	Direction of association vs. control	Key interpretation
Colchicine	ACS	Clinically significant infection; malignancy; diarrhea/GI upset	No increased risk of infection or malignancy; no significant increase in diarrhea/GI upset	Overall favorable safety profile in ACS; GI intolerance not significantly increased in pooled analyses
	Stable CAD	Clinically significant infection; malignancy; diarrhea/GI upset	No increased risk of infection or malignancy; increased risk of diarrhea/GI upset	GI intolerance remains the most common adverse effect in stable CAD
NSAIDs (non-aspirin)	ACS	Clinically significant infection; malignancy; diarrhea/GI upset	No increased risk of infection, malignancy, or GI upset	Findings based on sparse and predominantly historical data; cardiovascular safety concerns remain
	Stable CAD	Clinically significant infection; malignancy; diarrhea/GI upset	No increased risk of infection or malignancy; no significant increase in GI upset	Limited safety data; not recommended for secondary prevention
Corticosteroids	Stable CAD	Clinically significant infection; malignancy; GI upset	No significant increase in assessed adverse events	Evidence derived largely from older trials; long-term safety uncertain
Methotrexate	ACS	Diarrhea/GI upset	No significant increase	Limited data
	Stable CAD	Malignancy; diarrhea/GI upset	Increased risk of malignancy and GI upset	Safety signal limits clinical applicability
Darapladib	ACS	Malignancy; diarrhea/GI upset	No increased risk	Neutral safety signal
	Stable CAD	Diarrhea/GI upset	Reduced risk of GI upset	Clinical significance uncertain
Other agents (canakinumab, varespladib, losmapimod, pexelizumab, salsalate)	ACS/Stable CAD	Various	No consistent increase in adverse events	Limited power; heterogeneous reporting

ACS, acute coronary syndrome; CAD, coronary artery disease; GI, gastrointestinal.

## Discussion

We identified 41 RCTs comparing anti-inflammatory agents for the prevention of MACE in patients with CAD. Of these, 21 RCTs were included in the ACS network analysis and 20 RCTs were included in the stable CAD network. To the best of our knowledge, this is the most up-to-date and complete systematic review and network meta-analysis to evaluate the currently available evidence and summarize the benefits and harms of anti-inflammatory therapies in the prevention of MACE in patients with known CAD. Our findings align with prior reports on the efficacy of colchicine for this indication, but this analysis expands upon that knowledge and allows for the comparison of colchicine to other anti-inflammatory therapies. We have also provided detailed evidence networks (57) which allow for the visualization of available evidence on this topic. We have provided a summary of the evidence for anti-inflammatory therapies for patients in both the ACS setting and the stable CAD setting. Importantly, a substantial proportion of included trials were conducted prior to the widespread adoption of statins, contemporary antiplatelet therapy, and drug-eluting stents. While inclusion of these studies allowed for a comprehensive assessment of the anti-inflammatory literature, findings from earlier eras should be regarded as hypothesis-generating rather than directly practice-changing. Moreover, interpretation of indirect comparisons and treatment rankings is constrained by sparse network geometry, with many treatment nodes informed by single studies and connected predominantly through a common control.

In the ACS setting, colchicine was associated with a reduction in MACE relative to standard of care (no anti-inflammatory therapies). An apparent association between NSAID use and reduced MACE was also observed; however, this finding was driven by sparse and predominantly indirect evidence. Conversely, Varespladib was associated with an increased risk of MACE compared to standard of care. In the stable CAD setting, both corticosteroids and colchicine were associated with a reduction in MACE compared to standard of care. None of the investigated anti-inflammatory therapies were associated with an increased risk of MACE relative to the standard of care in the stable CAD setting. Importantly, in sensitivity analyses restricting the ACS network to more contemporary trials, the apparent association between NSAID use and reduced MACE was no longer observed, indicating that the signal was driven by an older study and may reflect historical context rather than a true protective effect. For stable CAD, NSAID use was associated with a significant reduction in MACE when it had not been in the main analysis.

Adverse therapy effects are discussed in further detail in the secondary outcomes section, in the Supplementary Material. To further elaborate, prednisone was not observed to result in increased rates of gastrointestinal bleeding, clinically significant glucose intolerance, or increased risk of infection. Meloxicam use was not associated with an increased risk of bleeding or impairment of the cardiovascular, pulmonary, renal, or autonomic nervous systems. Celecoxib was not associated with an increased risk of bleeding, but some patients discontinued it due to gastrointestinal discomfort, skin rashes, pruritis, or headache. Regarding colchicine, a variable proportion of patients complained of gastrointestinal intolerance due to diarrhea, nausea, or vomiting. As with any treatment, physicians should counsel their patients about the expected benefits and harms of potential therapies and weigh them when making decisions.

Guidelines have increasingly recommended colchicine for the secondary prevention of atherosclerotic cardiovascular disease. In both the ACS and stable CAD networks, colchicine was associated with a lower risk of MACE compared with control (ACS: OR 0.77, 95% CrI 0.62–0.95; Stable CAD: OR 0.65, 95% CrI 0.54–0.77). Interestingly the European and South American CV prevention guidelines have endorsed colchicine as an anti-inflammatory therapy for the prevention of MACE in adult patients with CAD (5, 6). Likewise, both the FDA and Health Canada have followed suit, and both have endorsed colchicine for use in adult patients with known CAD for the prevention of MACE (4). No other anti-inflammatory therapies have been recommended for the prevention of cardiovascular disease yet. Although our analysis identified signals suggesting reduced MACE with NSAIDs in ACS (OR 0.30, 95% CrI 0.11–0.74) and corticosteroids in stable CAD (OR 0.44, 95% CrI 0.26–0.72), these findings are based on sparse, indirect, and largely historical evidence and should not be interpreted as supportive of their clinical use for secondary prevention. Rather, these observations should be regarded as hypothesis-generating and reflective of older trial contexts rather than practice-changing evidence.

The present analysis must be interpreted in the context of historical data on anti-inflammatory agents. While our findings suggest potential benefits with certain agents like colchicine, it's crucial to acknowledge the uncertainty and even documented harm associated with other anti-inflammatory strategies in cardiovascular disease. Early enthusiasm for selective COX-2 inhibitors was tempered by evidence of increased MACE. More broadly, non-aspirin NSAIDs as a class have been linked to increased cardiovascular risk, with strong recommendations against their use in patients with established or high risk for CVD (58). This context highlights the importance of cautious interpretation and the need for rigorous safety assessments of any anti-inflammatory agent considered for long-term cardiovascular protection. Our findings suggest colchicine as a promising agent with sufficient evidence to support its use. Future research should prioritize identifying patient subgroups who are most likely to benefit from anti-inflammatory therapies while minimizing potential harms.

### Limitations

There are several limitations to our analysis. First, despite marked clinical and methodological heterogeneity among included trials, we employed a fixed-effects network meta-analysis. This decision was driven by sparse network connectivity, with most treatment comparisons informed by single trials, which rendered random-effects models unstable and uninterpretable. The use of fixed-effects models assumes a common underlying treatment effect and likely biases estimates toward statistical significance, necessitating cautious interpretation of all effect estimates and treatment rankings. Secondly, there was considerable variability in follow-up duration across studies, and the use of odds ratios rather than hazard ratios precluded accounting for differential follow-up time and censoring, which may have increased the likelihood of detecting treatment effects in trials with longer follow-up and influenced the magnitude of observed associations. Although sensitivity analyses restricted to longer follow-up partially mitigate concerns regarding time-horizon heterogeneity, pooling across trials with differing endpoint definitions and risk windows may obscure differential short-term vs. long-term effects. Accordingly, results should be interpreted as average treatment effects across heterogeneous clinical contexts rather than as precise estimates for specific temporal risk windows. Thirdly, many of the studies included in the study had a small sample size (less than 200 patients). Additionally, heterogeneity in background medical and interventional therapies across study eras limits the generalizability of our findings to contemporary practice. Key clinical variables, including stent type and revascularization strategy, were incompletely reported across trials, and follow-up duration and endpoint definitions varied substantially. Given sparse network geometry and the inability to perform network meta-regression, indirect comparisons should be interpreted as exploratory and hypothesis-generating rather than definitive. Although sensitivity analyses restricted to trials published from 2010 onward partially mitigate these concerns, confounding by era-related factors cannot be excluded, and violations of the transitivity assumption remain possible. Overestimation of treatment effect because of a small sample size should not be overlooked. These limitations should caution the use of the present study's findings for informing the role of anti-inflammatory agents for the secondary prevention of cardiovascular disease in patients with known CAD.

A majority of the data for corticosteroids was in patients who received bare-metal stents (BMS). A benefit may have been observed in this population because the corticosteroids could have affected the rates of in-stent restenosis. However, drug-eluting stents (DES) are predominantly being used at the time of this study. Future studies could consider re-examining the utility of corticosteroids in patients with DES.

Furthermore, another concern is publication bias, which occurs when studies with significant or positive findings are more likely to be published than those with non-significant or negative results. As a result, the evidence base available for analysis may overrepresent favorable outcomes, skewing the results and leading to erroneous conclusions. Furthermore, excluding unpublished or less accessible studies might make the findings less representative of real-world outcomes. Consequently, the review could favor interventions that seem effective only in select populations or under specific conditions. Moreover, underreporting of adverse events in unpublished studies can underestimate risks associated with certain interventions. Particular to network meta-analyses, publication bias can result in fewer or no connections for interventions with less favorable results. This distorts the relative ranking of treatments, as comparisons may favor interventions with disproportionately positive evidence. In addition, network meta-analyses combine direct and indirect evidence. If publication bias affects the direct evidence, it propagates through the network and compromises indirect comparisons, leading to biased treatment rankings. Publication bias was combatted in our study by performing risk of bias assessments and searching for unpublished studies, conference abstracts, trial registries, and grey literature.

Finally, network meta-analyses are most valuable when comparing multiple active treatments across different studies. In the present study, most comparisons are between a single anti-inflammatory agent and placebo/control, and only two anti-inflammatory drugs of limited clinical relevance in cardiology have dosing studies that create actual network connections [Anakinra (high and low dose) and Losmapimod (high and low dose)]. Importantly, most indirect comparisons were graded as low or very low confidence by CINeMA due to imprecision and heterogeneity, and these ratings meaningfully constrain the strength of inference that can be drawn from the network. Considering these limitations, this study serves to provide a comprehensive overview of anti-inflammatory agent treatments in CAD patients and provides hypothesis generating findings to inform future research in the field.

## Conclusions

Compared with standard of care, colchicine demonstrated the most consistent association with reduction in MACE across analyses in patients with ACS, whereas observed associations for NSAIDs were driven by sparse and predominantly indirect evidence. Varespladib was associated with an increased risk of MACE relative to standard of care. In patients with stable CAD, corticosteroids and colchicine were associated with a reduction in MACE compared to standard of care; however, these findings were informed largely by older trials and indirect comparisons and should be interpreted with caution. Overall, the results of this review suggest that anti-inflammatory therapies confer benefit in selected CAD populations, but the strength of comparative inference is limited by sparse network geometry and low or very low confidence in many treatment comparisons. These findings should be interpreted as hypothesis-generating rather than practice-changing and highlight the need for contemporary head-to-head randomized trials. Further comparative studies or network meta-regression analyses of patient-level data are needed to better define the relative efficacy and safety of anti-inflammatory strategies in these populations.

## Data Availability

The original contributions presented in the study are included in the article/Supplementary Material, further inquiries can be directed to the corresponding author.
