# Supplementary material for: Anti-inflammatory therapies to prevent cardiovascular events: systematic review and network meta-analysis of randomised controlled trials
Source: Front Cardiovasc Med. 2026 Mar 11;13:1717817. doi: 10.3389/fcvm.2026.1717817 (PMC13013507; doi:10.3389/fcvm.2026.1717817)
Supplement: Supplementary file 1 [file Datasheet1.docx]

**Supplementary Materials**

Table of Contents

[S1 - Supplementary Table 1. Medline Search 2](#_Toc219627037)

[S2 - Supplementary Table 2. Embase Search 7](#_Toc219627038)

[S3 - Supplementary Table 3. Cochrane Central Register of Controlled Trials Search 12](#_Toc219627039)

[S4 - Supplementary Table 4. ClinicalTrials.Gov Search 16](#_Toc219627040)

[S5 - Supplementary Table 5. World Health Organization International Clinical Trials Registry Platform (ICTRP) Search 17](#_Toc219627041)

[S6 - Supplementary Table 6. Europe PMC Search (https://europepmc.org/) 18](#_Toc219627042)

[S7 - Supplementary Table 7. List of Conferences Hand-searched 19](#_Toc219627043)

[S13 - Supplementary Table 13. Confidence in the results. 27](#_Toc219627044)

[S15 - Supplementary Table 15. Chronic CAD – MACE Evidence Summary Table 29](#_Toc219627045)

[S16 - Supplementary Table 16. ACS – MACE Evidence Summary Table for Studies with ≥30 Days of Follow Up and ≥30 Days of Treatment 30](#_Toc219627046)

[S17 - Supplementary Table 17. ACS – MACE Evidence Summary Table for Studies Published 2010 or Later with ≥30 Days of Follow Up and ≥30 Days of Treatment 31](#_Toc219627047)

[S18 - Supplementary Table 18. Chronic CAD – MACE Evidence Summary Table for Studies with ≥30 Days of Follow Up and ≥30 Days of Treatment 32](#_Toc219627048)

[S19 - Supplementary Table 19. Chronic CAD – MACE Evidence Summary Table for Studies Published 2010 or Later with ≥30 Days of Follow Up and ≥30 Days of Treatment 33](#_Toc219627049)

[S20 - Supplementary Figure 1. Inconsistency plot for stable CAD network. Shows posterior mean deviance of the individual data points from fitted consistency and inconsistency models. 34](#_Toc219627050)

[S21 - Supplementary Figure 2. Inconsistency plot for ACS network. Shows posterior mean deviance of the individual data points from fitted consistency and inconsistency models. 35](#_Toc219627051)

[**Secondary Outcomes** 36](#_Toc219627052)

# S1 - Supplementary Table 1. Medline Search

| **Database: Medline Search (Ovid MEDLINE® ALL 1946 to February 17, 2022)**  **Platform: Ovid**  **Date Searched:** **February 18, 2022** | | |
| --- | --- | --- |
| **#** | **Searches** | **Results** |
| 1 | exp Arteriosclerosis/ or Plaque, Atherosclerotic/ | 197274 |
| 2 | (atherosclero* or athero-sclero* or arteriosclero* or arterial-sclero* or arteriolosclero* or arteriolo-sclero* or vascular sclero* or ASCVD or ASCAD or intima plaque).ti,ab,kf,kw. | 184426 |
| 3 | Acute Coronary Syndrome/ or exp Coronary Disease/ | 243706 |
| 4 | ((coronary adj3 (arter* or stenos* or disease? or disorder? or syndrom?)) or CAD or SCAD).ti,ab,kf,kw. | 328530 |
| 5 | exp Percutaneous Coronary Intervention/ or exp Myocardial Revascularization/ | 116164 |
| 6 | (((aortocoronary or aorto-coronary or coronary) adj3 bypass*) or CABG).ti,ab,kf,kw. | 57086 |
| 7 | exp endarterectomy/ or exp thrombectomy/ or exp Atherectomy/ or exp Embolectomy/ | 30081 |
| 8 | (angioplast* or atherectom* or endarterectom* or thrombectom* or thromboendarterectom* or thrombo-endarterectom* or PCI or PTCA or (Percutaneous adj3 (intervent* or revascular*))).ti,ab,kf,kw. | 127048 |
| 9 | or/1-8 | 704058 |
| 10 | exp Anti-Inflammatory Agents, Non-Steroidal/ | 208246 |
| 11 | (((non-steroidal or nonsteroidal) adj2 (anti-inflammatory or antiinflammatory or analg?esic*)) or NSAID*).ti,ab,kf,kw. | 50432 |
| 12 | (acalabrutinib* or aceclofenac* or acemetacin* or acetaminosalol* or acetylsalicylate* or acetylsalicylic* or aclantate* or actarit* or adalimumab* or afasevikumab* or afimetoran* or alclofenac* or aldafermin* or alminoprofen* or aloxiprin* or amfenac* or aminophenazone* or aminosalicylic* or amlitelimab* or amlodipine* or ampiroxicam* or amtolmetin guacil* or anirolac* or antiflammin* or apadenoson* or apremilast* or araprofen* or ascription* or asivatrep* or astegolimab* or atibuclimab* or atliprofen* or aviptadil* or azathioprine* or azelaic acid* or bakeprofen* or balsalazide* or bardoxolone* or bardoxolone methyl* or belumosudil* or bendazac* or benorilate* or benoxaprofen* or bermoprofen* or bimosiamose* or brazikumab* or brensocatib* or brimonidine* or bromfenac* or broperamole* or bucloxic acid* or bucolome* or bufexamac* or butibufen* or camobucol* or carbasalate* or carotegrast* or carprofen* or cedirogant* or celecoxib* or cibinetide* or cicloprofen* or cimicoxib* or cinmetacin* or cinnoxicam* or clidanac* or clofezone* or clonixin* or clonixin lysine* or cloximate* or crisaborole*).ti,ab,kf,kw. | 59208 |
| 13 | (dagrocorat* or danicopan* or dapansutrile* or dapatifagene navolactibac* or darbufelone* or daxdilimab* or dazodalibep* or dehydrozingerone* or demethoxycurcumin* or deracoxib* or deucravacitinib* or dexibuprofen* or dexketoprofen* or dexpemedolac* or diclofenac* or didemethoxycurcumin* or diflunisal* or diftalone* or dimethyl fumarate* or dimethyl sulfoxide* or diphenpyramide* or ditazole* or droxicam* or duometacin* or ebdarokimab* or ebselen* or edasalonexent* or efruxifermin* or elsibucol* or emavusertib* or emorfazone* or emvododstat* or endolac* or enfenamic acid* or enflicoxib* or enpatoran* or epirizole* or etodolac* or etofenamate* or etoricoxib* or evobrutinib* or felbinac* or fenamic acid* or fenbufen* or fenclofenac* or fenclozic acid* or fendosal* or fenflumizole* or fenoprofen* or fentiazac* or fepradinol* or feprazone* or firategrast* or firocoxib* or flobufen* or flosulide* or flufenamate aluminum* or flufenamic acid* or flunixin* or flunoxaprofen* or fluproquazone* or flurbiprofen* or flutiazin* or fosdagrocorat* or fosfosal* or furaprofen* or furcloprofen* or furobufen* or furofenac* or fuzapladib*).ti,ab,kf,kw. | 36777 |
| 14 | (glucametacin* or gluconate zinc* or guacetisal* or guaimesal* or gusacitinib* or hydroxychloroquine* or ibrigampar* or ibufenac* or ibuprofen* or ibuproxam* or icoduline* or icosapentaenoic acid* or iguratimod* or ilonidap* or imidazole salicylate* or imisopasem manganese* or imsidolimab* or incyclinide* or indameth* or indometacin* or indoprofen* or ipsalazide* or iptacopan* or isecarosmab* or isofezolac* or isonixin* or isoxepac* or isoxicam* or itepekimab* or kebuzone* or ketoprofen* or ketoprofen lysine* or ketorolac* or lazertinib* or lazucirnon* or leflunomide* or lenabasum* or licofelone* or lifitegrast* or lirentelimab* or lobuprofen* or lonazolac* or lorecivivint* or lornoxicam* or losmiprofen* or loxoprofen* or lumiracoxib* or lusvertikimab* or lyprinol* or lysine acetylsalicylate*).ti,ab,kf,kw. | 33747 |
| 15 | (mabuprofen* or magnesium salicylate* or manoalide* or mapracorat* or mavacoxib* or meclofenam* or mefenamic acid* or meloxicam* or melrilimab* or mesalazine* or methotrexate* or metiazinic acid* or metoxibutropate* or milategrast* or mipragoside* or mirococept* or miroprofen* or mivavotinib* or mofebutazone* or mofezolac* or mongersen* or morazone* or morniflumate* or mosedipimod* or nabumetone* or nangibotide* or naproxcinod* or naproxen* or navamepent* or nepafenac* or neurofenac* or neurotropin* or nictindole* or niflumic acid* or nimesulide* or ocarocoxib* or odalprofen* or olsalazine* or ordesekimab* or ormeloxifene* or orpanoxin* or otenaproxesul* or oxaceprol* or oxametacin* or oxaprazine* or oxaprozin* or oxicam derivative* or oxindanac* or oxyphenbutazone*).ti,ab,kf,kw. | 62386 |
| 16 | (palifermin* or parcetasal* or parecoxib* or pelubiprofen* or pemedolac* or perisoxal* or phenylbutazone* or phenylbutazone megallate* or picolamine salicylate* or piketoprofen* or pimeprofen* or pipebuzone* or piproxen* or pirazolac* or pirfenidone* or piroxicam* or piroxicam beta cyclodextrin* or pirprofen* or plonmarlimab* or plozalizumab* or polmacoxib* or pralnacasan* or pranoprofen* or prinomide* or prinomide triethanolamine* or proglumetacin* or proquazone* or pyrazinobutazone* or quellor* or rapamycin* or rasagiline* or ravagalimab* or relfovetmab* or reltecimod* or remestemcel L* or resatorvid* or rimacalib* or rimazolium* or risankizumab* or robenacoxib* or rofecoxib* or romazarit* or rosiptor* or rosmarinic acid* or rovazolac* or rozibafusp alfa* or ruxolitinib* or salazosulfapyridine* or salicylic acid* or salnacedin* or salsalate* or satralizumab* or scalaradial* or semapimod* or semorinemab* or sibofimloc* or simufilam* or sudoxicam* or sulfosalicylate samarium* or sulindac* or suprofen* or suxibuzone*).ti,ab,kf,kw. | 67671 |
| 17 | (talniflumate* or tapinarof* or tazofelone* or telazorlimab* or tenidap* or tenosal* or tenosiprol* or tenoxicam* or tepoxalin* or teriflunomide* or tesnatilimab* or tiaprofenic acid* or tiaramide* or tilmacoxib* or tilnoprofen arbamel* or tilomisole* or timegadine* or tioxamast* or tioxaprofen* or tirnovetmab* or tolebrutinib* or tolfenamic acid* or tolmetin* or tomaralimab* or tomicorat* or torudokimab* or tozorakimab* or tralokinumab* or tribuzone* or triethanolamine salicylate* or tropesin* or tryptamide* or ufenamate* or valategrast* or valdecoxib* or valerylsalicylic acid* or vasoactive intestinal polypeptide* or vedaprofen* or velsecorat* or vemircopan* or verramed* or vilobelimab* or vixarelimab* or ximoprofen* or zabedosertib* or zaloglanstat* or zaltoprofen* or zaurategrast* or zidometacin* or zinc salicylate* or zoliprofen* or zomepirac*).ti,ab,kf,kw. | 8947 |
| 18 | exp Colchicine/ | 15610 |
| 19 | (Colbenemid* or Colchichin* or colchicum* or colchily* or colchineos* or colchimedio* or colchiquim* or colchisol* or colchysat* or colcin* or colcrys* or colctab* or colgout* or colrefuz* or colsaloid* or Condylon* or gloperba* or goutichine* or goutnil* or kolkicin* or kolkisin* or mitigare* or tolchicine*).ti,ab,kf,kw. | 340 |
| 20 | (64-86-8* or SML2Y3J35T*).rn. | 14615 |
| 21 | Prednisone/ | 40606 |
| 22 | (adasone* or acsis* or ancortone* apo-prednisone* or bicortone* or biocortone* or cartancyl* or colisone* or Cortan* or cortancyl* or cortidelt* or cortiprex* or cotone* or cutason* or dacorten* or dacortin* or decortancyl* or decortin* or de-cortisyl* or decortisyl* or dihydrocortisone* or dihydrocortisone* or dekortin or dellacort* or deltacorten* or delta-cortelan* or delta-cortisone* or deltacortisone* or deltacortone* or delta-dome* or delta-prenovis* or deltasone* or delitisone* or deltison* or deltra* or diadreson* or di-adreson* or drazone* or econosone* or encorton* or encortone* or enkorton* or enkortolon* or fernisone* or fiasone* or hostacortin* or insone* or incocortyl* or juvason* or kortancyl* or liquid pred* or lodotra* or lodtra* or lisacort* or me-korti* or meprison* or metacortandracin* or Meticorten* or meticortine* or nisona* or nizon* or novoprednisone* or nurison* or Orasone* or orisane* or panafcort* or paracort* or panasol* or parmenison* or pehacort* or predeltin* or precort* or precortal* or prednicen* or prednicorm* or prednicort* or prednicot* or predni tablinen* or prednidib* or prednilonga* or predniment* or prednison* or prednitone* or prednizon* or prednovister* or presone* or pronison* or pronizon* or pulmison* or rayos* or rectodelt* or rectrocortine* or servisone* or sone$2 or steerometz* or sterapred* or supercortil* or ultracorten* or urtilone* or winpred* or wojtab* or zenadrid*).ti,ab,kf,kw. | 32967 |
| 23 | (53-03-2 or VB0R961HZT).rn. | 40606 |
| 24 | Methotrexate/ | 39859 |
| 25 | (abitextrate* or abitrexate* or amethopterin* or amethopterine* or ametopterine* or antifolan* or biotrexate* or brimexate* or canceren* or emtexate* or emthexat* or emthexate* or emtrexate* or enthexate* or farmitrexat* or farmotrex* or fauldexato* or folex* or ifamet* or imeth$2 or intradose MTX or jylamvo* or lantarel* or ledertrexate* or lumexon* or maxtrex* or medsatrexate* or metatrexan* or metex* or methoblastin* or methohexate* or methotrate* or methotrexat* or methylaminopterin* or metical* or metoject* or metotressato* or metothrexate* or metotrexat* or metotrexin* or metrex* or metrotex* or mexate* or neotrexate* or nordimet* or novatrex* or otrexup* or rasuvo* or reditrex* or reumatrex* or rheumatrex* or texate* or texorate* or tremetex* or trexall* or trexeron* or trixilem* or xaken* or xatmep* or zexate*).ti,ab,kf,kw. | 45598 |
| 26 | (59-05-2 or YL5FZ2Y5U1).rn. | 39859 |
| 27 | Interleukin-1beta/ | 26436 |
| 28 | (ACZ-885* or ACZ885* or Canakinumab* or ilaris*).ti,ab,kf,kw. | 814 |
| 29 | (914613-48-2 or 37CQ2C7X93).rn. | 498 |
| 30 | Antibodies, Monoclonal, Humanized/ | 48944 |
| 31 | (pexelizumab or "h5G1.1-SC*").ti,ab,kf,kw. | 95 |
| 32 | (219685-93-5 or CHZ6OLQ3UU).rn. | 70 |
| 33 | Interleukin 1 Receptor Antagonist Protein/ | 5581 |
| 34 | (Anakinra* or Antril* or Kineret* or il-1ra* or (interleukin 1 receptor adj1 (antagonist or block* or inhibit*))).ti,ab,kf,kw. | 9416 |
| 35 | (143090-92-0 or 9013DUQ28K).rn. | 0 |
| 36 | (agi-1067* or agi1067* or agz-1067* or agz1067* or probucol succinate* or Succinobucol*).ti,ab,kf,kw. | 58 |
| 37 | (216167-82-7 or J1J54V24R4).rn. | 52 |
| 38 | (ftx-1821* or ftx1821* or gsk-856553* or gsk856553* or gw-856553* or gw856553* or Losmapimod* or sb-856553* or sb856553*).ti,ab,kf,kw. | 52 |
| 39 | (F2DQF16BXE or 585543-15-3).rn. | 0 |
| 40 | (lc1004* or lc-1004* or inclacumab* or ro4905417* or ro-4905417*).ti,ab,kf,kw. | 10 |
| 41 | (1256258-86-2 or A6734I702L).rn. | 6 |
| 42 | (Atreleuton* or a85761* or a-85761* or abt761* or abt-761* or via-2291* or via2291*).ti,ab,kf,kw. | 25 |
| 43 | (U301T88E1M or 154355-76-7).rn. | 20 |
| 44 | (darapladib* or sb-480848* or sb480848*).ti,ab,kf,kw. | 143 |
| 45 | (356057-34-6 or UI1U1MYH09).rn. | 91 |
| 46 | (Varespladib* or Varepladib* or ly315920* or ly-315920* or s-5920* or s5920*).ti,ab,kf,kw. | 101 |
| 47 | (2Q3P98DATH or 172732-68-2).rn. | 52 |
| 48 | or/10-47 | 554747 |
| 49 | randomized controlled trial.pt. | 558953 |
| 50 | controlled clinical trial.pt. | 94700 |
| 51 | randomized.ab. | 551166 |
| 52 | placebo.ab. | 225750 |
| 53 | clinical trials as topic.sh. | 199220 |
| 54 | randomly.ab. | 376371 |
| 55 | trial.ti. | 256986 |
| 56 | 49 or 50 or 51 or 52 or 53 or 54 or 55 | 1427763 |
| 57 | exp animals/ not humans.sh. | 4960445 |
| 58 | 56 not 57 | 1313386 |
| 59 | 9 and 48 and 58 | 3919 |

# S2 - Supplementary Table 2. Embase Search

| **Database: Embase Classic+Embase 1947 to 2022 February 17**  **Platform: Ovid**  **Date Searched: February 18, 2022** | | |
| --- | --- | --- |
| **#** | **Searches** | **Results** |
| 1 | exp arteriosclerosis/ | 293364 |
| 2 | (atherosclero* or athero-sclero* or arteriosclero* or arterial-sclero* or arteriolosclero* or arteriolo-sclero* or vascular sclero* or ASCVD or ASCAD or intima plaque).ti,ab,kf,kw. | 276214 |
| 3 | exp coronary artery disease/ | 375951 |
| 4 | ((coronary adj3 (arter* or stenos* or disease? or disorder? or syndrom?)) or CAD or SCAD).ti,ab,kf,kw. | 494904 |
| 5 | exp interventional cardiovascular procedure/ or coronary artery bypass graft/ or exp coronary artery surgery/ | 268451 |
| 6 | (((aortocoronary or aorto-coronary or coronary) adj3 bypass*) or CABG).ti,ab,kf,kw. | 84156 |
| 7 | exp atherectomy/ or exp endarterectomy/ or exp embolectomy/ or exp thrombectomy/ | 73525 |
| 8 | (angioplast* or atherectom* or endarterectom* or thrombectom* or thromboendarterectom* or thrombo-endarterectom* or PCI or PTCA or (Percutaneous adj3 (intervent* or revascular*))).ti,ab,kf,kw. | 210906 |
| 9 | or/1-8 | 1092932 |
| 10 | exp *nonsteroid antiinflammatory agent/ | 335168 |
| 11 | (((non-steroidal or nonsteroidal) adj2 (anti-inflammatory or antiinflammatory or analg?esic*)) or NSAID*).ti,ab,kf,kw. | 80348 |
| 12 | (acalabrutinib* or aceclofenac* or acemetacin* or acetaminosalol* or acetylsalicylate* or acetylsalicylic* or aclantate* or actarit* or adalimumab* or afasevikumab* or afimetoran* or alclofenac* or aldafermin* or alminoprofen* or aloxiprin* or amfenac* or aminophenazone* or aminosalicylic* or amlitelimab* or amlodipine* or ampiroxicam* or amtolmetin guacil* or anirolac* or antiflammin* or apadenoson* or apremilast* or araprofen* or ascription* or asivatrep* or astegolimab* or atibuclimab* or atliprofen* or aviptadil* or azathioprine* or azelaic acid* or bakeprofen* or balsalazide* or bardoxolone* or bardoxolone methyl* or belumosudil* or bendazac* or benorilate* or benoxaprofen* or bermoprofen* or bimosiamose* or brazikumab* or brensocatib* or brimonidine* or bromfenac* or broperamole* or bucloxic acid* or bucolome* or bufexamac* or butibufen* or camobucol* or carbasalate* or carotegrast* or carprofen* or cedirogant* or celecoxib* or cibinetide* or cicloprofen* or cimicoxib* or cinmetacin* or cinnoxicam* or clidanac* or clofezone* or clonixin* or clonixin lysine* or cloximate* or crisaborole*).ti,ab,kf,kw. | 100591 |
| 13 | (dagrocorat* or danicopan* or dapansutrile* or dapatifagene navolactibac* or darbufelone* or daxdilimab* or dazodalibep* or dehydrozingerone* or demethoxycurcumin* or deracoxib* or deucravacitinib* or dexibuprofen* or dexketoprofen* or dexpemedolac* or diclofenac* or didemethoxycurcumin* or diflunisal* or diftalone* or dimethyl fumarate* or dimethyl sulfoxide* or diphenpyramide* or ditazole* or droxicam* or duometacin* or ebdarokimab* or ebselen* or edasalonexent* or efruxifermin* or elsibucol* or emavusertib* or emorfazone* or emvododstat* or endolac* or enfenamic acid* or enflicoxib* or enpatoran* or epirizole* or etodolac* or etofenamate* or etoricoxib* or evobrutinib* or felbinac* or fenamic acid* or fenbufen* or fenclofenac* or fenclozic acid* or fendosal* or fenflumizole* or fenoprofen* or fentiazac* or fepradinol* or feprazone* or firategrast* or firocoxib* or flobufen* or flosulide* or flufenamate aluminum* or flufenamic acid* or flunixin* or flunoxaprofen* or fluproquazone* or flurbiprofen* or flutiazin* or fosdagrocorat* or fosfosal* or furaprofen* or furcloprofen* or furobufen* or furofenac* or fuzapladib*).ti,ab,kf,kw. | 49581 |
| 14 | (glucametacin* or gluconate zinc* or guacetisal* or guaimesal* or gusacitinib* or hydroxychloroquine* or ibrigampar* or ibufenac* or ibuprofen* or ibuproxam* or icoduline* or icosapentaenoic acid* or iguratimod* or ilonidap* or imidazole salicylate* or imisopasem manganese* or imsidolimab* or incyclinide* or indameth* or indometacin* or indoprofen* or ipsalazide* or iptacopan* or isecarosmab* or isofezolac* or isonixin* or isoxepac* or isoxicam* or itepekimab* or kebuzone* or ketoprofen* or ketoprofen lysine* or ketorolac* or lazertinib* or lazucirnon* or leflunomide* or lenabasum* or licofelone* or lifitegrast* or lirentelimab* or lobuprofen* or lonazolac* or lorecivivint* or lornoxicam* or losmiprofen* or loxoprofen* or lumiracoxib* or lusvertikimab* or lyprinol* or lysine acetylsalicylate*).ti,ab,kf,kw. | 52974 |
| 15 | (mabuprofen* or magnesium salicylate* or manoalide* or mapracorat* or mavacoxib* or meclofenam* or mefenamic acid* or meloxicam* or melrilimab* or mesalazine* or methotrexate* or metiazinic acid* or metoxibutropate* or milategrast* or mipragoside* or mirococept* or miroprofen* or mivavotinib* or mofebutazone* or mofezolac* or mongersen* or morazone* or morniflumate* or mosedipimod* or nabumetone* or nangibotide* or naproxcinod* or naproxen* or navamepent* or nepafenac* or neurofenac* or neurotropin* or nictindole* or niflumic acid* or nimesulide* or ocarocoxib* or odalprofen* or olsalazine* or ordesekimab* or ormeloxifene* or orpanoxin* or otenaproxesul* or oxaceprol* or oxametacin* or oxaprazine* or oxaprozin* or oxicam derivative* or oxindanac* or oxyphenbutazone*).ti,ab,kf,kw. | 102431 |
| 16 | (palifermin* or parcetasal* or parecoxib* or pelubiprofen* or pemedolac* or perisoxal* or phenylbutazone* or phenylbutazone megallate* or picolamine salicylate* or piketoprofen* or pimeprofen* or pipebuzone* or piproxen* or pirazolac* or pirfenidone* or piroxicam* or piroxicam beta cyclodextrin* or pirprofen* or plonmarlimab* or plozalizumab* or polmacoxib* or pralnacasan* or pranoprofen* or prinomide* or prinomide triethanolamine* or proglumetacin* or proquazone* or pyrazinobutazone* or quellor* or rapamycin* or rasagiline* or ravagalimab* or relfovetmab* or reltecimod* or remestemcel L* or resatorvid* or rimacalib* or rimazolium* or risankizumab* or robenacoxib* or rofecoxib* or romazarit* or rosiptor* or rosmarinic acid* or rovazolac* or rozibafusp alfa* or ruxolitinib* or salazosulfapyridine* or salicylic acid* or salnacedin* or salsalate* or satralizumab* or scalaradial* or semapimod* or semorinemab* or sibofimloc* or simufilam* or sudoxicam* or sulfosalicylate samarium* or sulindac* or suprofen* or suxibuzone*).ti,ab,kf,kw. | 89655 |
| 17 | (talniflumate* or tapinarof* or tazofelone* or telazorlimab* or tenidap* or tenosal* or tenosiprol* or tenoxicam* or tepoxalin* or teriflunomide* or tesnatilimab* or tiaprofenic acid* or tiaramide* or tilmacoxib* or tilnoprofen arbamel* or tilomisole* or timegadine* or tioxamast* or tioxaprofen* or tirnovetmab* or tolebrutinib* or tolfenamic acid* or tolmetin* or tomaralimab* or tomicorat* or torudokimab* or tozorakimab* or tralokinumab* or tribuzone* or triethanolamine salicylate* or tropesin* or tryptamide* or ufenamate* or valategrast* or valdecoxib* or valerylsalicylic acid* or vasoactive intestinal polypeptide* or vedaprofen* or velsecorat* or vemircopan* or verramed* or vilobelimab* or vixarelimab* or ximoprofen* or zabedosertib* or zaloglanstat* or zaltoprofen* or zaurategrast* or zidometacin* or zinc salicylate* or zoliprofen* or zomepirac*).ti,ab,kf,kw. | 11952 |
| 18 | *colchicine/ | 12844 |
| 19 | (Colbenemid* or Colchichin* or colchicum* or colchily* or colchineos* or colchimedio* or colchiquim* or colchisol* or colchysat* or colcin* or colcrys* or colctab* or colgout* or colrefuz* or colsaloid* or Condylon* or gloperba* or goutichine* or goutnil* or kolkicin* or kolkisin* or mitigare* or tolchicine*).ti,ab,kf,kw. | 473 |
| 20 | (64-86-8* or SML2Y3J35T*).rn. | 34251 |
| 21 | *prednisone/ | 43283 |
| 22 | (adasone* or acsis* or ancortone* apo-prednisone* or bicortone* or biocortone* or cartancyl* or colisone* or Cortan* or cortancyl* or cortidelt* or cortiprex* or cotone* or cutason* or dacorten* or dacortin* or decortancyl* or decortin* or de-cortisyl* or decortisyl* or dihydrocortisone* or dihydrocortisone* or dekortin or dellacort* or deltacorten* or delta-cortelan* or delta-cortisone* or deltacortisone* or deltacortone* or delta-dome* or delta-prenovis* or deltasone* or delitisone* or deltison* or deltra* or diadreson* or di-adreson* or drazone* or econosone* or encorton* or encortone* or enkorton* or enkortolon* or fernisone* or fiasone* or hostacortin* or insone* or incocortyl* or juvason* or kortancyl* or liquid pred* or lodotra* or lodtra* or lisacort* or me-korti* or meprison* or metacortandracin* or Meticorten* or meticortine* or nisona* or nizon* or novoprednisone* or nurison* or Orasone* or orisane* or panafcort* or paracort* or panasol* or parmenison* or pehacort* or predeltin* or precort* or precortal* or prednicen* or prednicorm* or prednicort* or prednicot* or predni tablinen* or prednidib* or prednilonga* or predniment* or prednison* or prednitone* or prednizon* or prednovister* or presone* or pronison* or pronizon* or pulmison* or rayos* or rectodelt* or rectrocortine* or servisone* or sone$2 or steerometz* or sterapred* or supercortil* or ultracorten* or urtilone* or winpred* or wojtab* or zenadrid*).ti,ab,kf,kw. | 61627 |
| 23 | (53-03-2 or VB0R961HZT).rn. | 178929 |
| 24 | *methotrexate/ | 53338 |
| 25 | (abitextrate* or abitrexate* or amethopterin* or amethopterine* or ametopterine* or antifolan* or biotrexate* or brimexate* or canceren* or emtexate* or emthexat* or emthexate* or emtrexate* or enthexate* or farmitrexat* or farmotrex* or fauldexato* or folex* or ifamet* or imeth$2 or intradose MTX or jylamvo* or lantarel* or ledertrexate* or lumexon* or maxtrex* or medsatrexate* or metatrexan* or metex* or methoblastin* or methohexate* or methotrate* or methotrexat* or methylaminopterin* or metical* or metoject* or metotressato* or metothrexate* or metotrexat* or metotrexin* or metrex* or metrotex* or mexate* or neotrexate* or nordimet* or novatrex* or otrexup* or rasuvo* or reditrex* or reumatrex* or rheumatrex* or texate* or texorate* or tremetex* or trexall* or trexeron* or trixilem* or xaken* or xatmep* or zexate*).ti,ab,kf,kw. | 78910 |
| 26 | (59-05-2 or YL5FZ2Y5U1).rn. | 180823 |
| 27 | canakinumab/ | 3930 |
| 28 | (ACZ-885* or ACZ885* or Canakinumab* or ilaris*).ti,ab,kf,kw. | 1881 |
| 29 | (914613-48-2 or 37CQ2C7X93).rn. | 2417 |
| 30 | pexelizumab/ | 360 |
| 31 | (pexelizumab or "h5G1.1-SC*").ti,ab,kf,kw. | 112 |
| 32 | (219685-93-5 or CHZ6OLQ3UU).rn. | 355 |
| 33 | anakinra/ | 4590 |
| 34 | (Anakinra* or Antril* or Kineret* or il-1ra* or (interleukin 1 receptor adj1 (antagonist or block* or inhibit*))).ti,ab,kf,kw. | 14433 |
| 35 | (143090-92-0 or 9013DUQ28K).rn. | 7368 |
| 36 | succinobucol/ | 75 |
| 37 | (agi-1067* or agi1067* or agz-1067* or agz1067* or probucol succinate* or Succinobucol*).ti,ab,kf,kw. | 76 |
| 38 | (216167-82-7 or J1J54V24R4).rn. | 152 |
| 39 | losmapimod/ | 207 |
| 40 | (ftx-1821* or ftx1821* or gsk-856553* or gsk856553* or gw-856553* or gw856553* or Losmapimod* or sb-856553* or sb856553*).ti,ab,kf,kw. | 83 |
| 41 | (F2DQF16BXE or 585543-15-3).rn. | 201 |
| 42 | inclacumab/ | 50 |
| 43 | (lc1004* or lc-1004* or inclacumab* or ro4905417* or ro-4905417*).ti,ab,kf,kw. | 20 |
| 44 | (1256258-86-2 or A6734I702L).rn. | 46 |
| 45 | atreleuton/ | 161 |
| 46 | (Atreleuton* or a85761* or a-85761* or abt761* or abt-761* or via-2291* or via2291*).ti,ab,kf,kw. | 37 |
| 47 | (U301T88E1M or 154355-76-7).rn. | 156 |
| 48 | darapladib/ | 411 |
| 49 | (darapladib* or sb-480848* or sb480848*).ti,ab,kf,kw. | 207 |
| 50 | (356057-34-6 or UI1U1MYH09).rn. | 376 |
| 51 | varespladib/ | 233 |
| 52 | (Varespladib* or Varepladib* or ly315920* or ly-315920* or s-5920* or s5920*).ti,ab,kf,kw. | 125 |
| 53 | (2Q3P98DATH or 172732-68-2).rn. | 218 |
| 54 | or/10-53 | 870554 |
| 55 | crossover procedure/ | 69817 |
| 56 | double blind procedure/ | 194935 |
| 57 | exp randomized controlled trial/ | 700059 |
| 58 | single blind procedure/ | 45231 |
| 59 | (random$ or factorial$ or crossover$ or cross over$ or cross-over$ or placebo$ or (doubl$ adj blind$) or (singl$ adj blind$) or assign$ or allocat$ or volunteer$).mp. | 2947243 |
| 60 | 55 or 56 or 57 or 58 or 59 | 2947906 |
| 61 | exp animal/ | 30333319 |
| 62 | exp human/ | 24637922 |
| 63 | 61 not 62 | 5695397 |
| 64 | 60 not 63 | 2682255 |
| 65 | 9 and 54 and 64 | 7316 |

# S3 - Supplementary Table 3. Cochrane Central Register of Controlled Trials Search

| **Database: EBM Reviews - Cochrane Central Register of Controlled Trials January 2022**  **Platform: Ovid**  **Date Searched: February 18, 2022** | | |
| --- | --- | --- |
| **#** | **Searches** | **Results** |
| 1 | exp Arteriosclerosis/ or Plaque, Atherosclerotic/ | 11471 |
| 2 | (atherosclero* or athero-sclero* or arteriosclero* or arterial-sclero* or arteriolosclero* or arteriolo-sclero* or vascular sclero* or ASCVD or ASCAD or intima plaque).ti,ab. | 12951 |
| 3 | Acute Coronary Syndrome/ or exp Coronary Disease/ | 16154 |
| 4 | ((coronary adj3 (arter* or stenos* or disease? or disorder? or syndrom?)) or CAD or SCAD).ti,ab. | 42037 |
| 5 | exp Percutaneous Coronary Intervention/ or exp Myocardial Revascularization/ | 11573 |
| 6 | (((aortocoronary or aorto-coronary or coronary) adj3 bypass*) or CABG).ti,ab. | 12770 |
| 7 | exp endarterectomy/ or exp thrombectomy/ or exp Atherectomy/ or exp Embolectomy/ | 1084 |
| 8 | (angioplast* or atherectom* or endarterectom* or thrombectom* or thromboendarterectom* or thrombo-endarterectom* or PCI or PTCA or (Percutaneous adj3 (intervent* or revascular*))).ti,ab. | 22547 |
| 9 | or/1-8 | 73860 |
| 10 | exp Anti-Inflammatory Agents, Non-Steroidal/ | 20078 |
| 11 | (((non-steroidal or nonsteroidal) adj2 (anti-inflammatory or antiinflammatory or analg?esic*)) or NSAID*).ti,ab. | 11105 |
| 12 | (acalabrutinib* or aceclofenac* or acemetacin* or acetaminosalol* or acetylsalicylate* or acetylsalicylic* or aclantate* or actarit* or adalimumab* or afasevikumab* or afimetoran* or alclofenac* or aldafermin* or alminoprofen* or aloxiprin* or amfenac* or aminophenazone* or aminosalicylic* or amlitelimab* or amlodipine* or ampiroxicam* or amtolmetin guacil* or anirolac* or antiflammin* or apadenoson* or apremilast* or araprofen* or ascription* or asivatrep* or astegolimab* or atibuclimab* or atliprofen* or aviptadil* or azathioprine* or azelaic acid* or bakeprofen* or balsalazide* or bardoxolone* or bardoxolone methyl* or belumosudil* or bendazac* or benorilate* or benoxaprofen* or bermoprofen* or bimosiamose* or brazikumab* or brensocatib* or brimonidine* or bromfenac* or broperamole* or bucloxic acid* or bucolome* or bufexamac* or butibufen* or camobucol* or carbasalate* or carotegrast* or carprofen* or cedirogant* or celecoxib* or cibinetide* or cicloprofen* or cimicoxib* or cinmetacin* or cinnoxicam* or clidanac* or clofezone* or clonixin* or clonixin lysine* or cloximate* or crisaborole*).ti,ab. | 17081 |
| 13 | (dagrocorat* or danicopan* or dapansutrile* or dapatifagene navolactibac* or darbufelone* or daxdilimab* or dazodalibep* or dehydrozingerone* or demethoxycurcumin* or deracoxib* or deucravacitinib* or dexibuprofen* or dexketoprofen* or dexpemedolac* or diclofenac* or didemethoxycurcumin* or diflunisal* or diftalone* or dimethyl fumarate* or dimethyl sulfoxide* or diphenpyramide* or ditazole* or droxicam* or duometacin* or ebdarokimab* or ebselen* or edasalonexent* or efruxifermin* or elsibucol* or emavusertib* or emorfazone* or emvododstat* or endolac* or enfenamic acid* or enflicoxib* or enpatoran* or epirizole* or etodolac* or etofenamate* or etoricoxib* or evobrutinib* or felbinac* or fenamic acid* or fenbufen* or fenclofenac* or fenclozic acid* or fendosal* or fenflumizole* or fenoprofen* or fentiazac* or fepradinol* or feprazone* or firategrast* or firocoxib* or flobufen* or flosulide* or flufenamate aluminum* or flufenamic acid* or flunixin* or flunoxaprofen* or fluproquazone* or flurbiprofen* or flutiazin* or fosdagrocorat* or fosfosal* or furaprofen* or furcloprofen* or furobufen* or furofenac* or fuzapladib*).ti,ab. | 8122 |
| 14 | (glucametacin* or gluconate zinc* or guacetisal* or guaimesal* or gusacitinib* or hydroxychloroquine* or ibrigampar* or ibufenac* or ibuprofen* or ibuproxam* or icoduline* or icosapentaenoic acid* or iguratimod* or ilonidap* or imidazole salicylate* or imisopasem manganese* or imsidolimab* or incyclinide* or indameth* or indometacin* or indoprofen* or ipsalazide* or iptacopan* or isecarosmab* or isofezolac* or isonixin* or isoxepac* or isoxicam* or itepekimab* or kebuzone* or ketoprofen* or ketoprofen lysine* or ketorolac* or lazertinib* or lazucirnon* or leflunomide* or lenabasum* or licofelone* or lifitegrast* or lirentelimab* or lobuprofen* or lonazolac* or lorecivivint* or lornoxicam* or losmiprofen* or loxoprofen* or lumiracoxib* or lusvertikimab* or lyprinol* or lysine acetylsalicylate*).ti,ab. | 11607 |
| 15 | (mabuprofen* or magnesium salicylate* or manoalide* or mapracorat* or mavacoxib* or meclofenam* or mefenamic acid* or meloxicam* or melrilimab* or mesalazine* or methotrexate* or metiazinic acid* or metoxibutropate* or milategrast* or mipragoside* or mirococept* or miroprofen* or mivavotinib* or mofebutazone* or mofezolac* or mongersen* or morazone* or morniflumate* or mosedipimod* or nabumetone* or nangibotide* or naproxcinod* or naproxen* or navamepent* or nepafenac* or neurofenac* or neurotropin* or nictindole* or niflumic acid* or nimesulide* or ocarocoxib* or odalprofen* or olsalazine* or ordesekimab* or ormeloxifene* or orpanoxin* or otenaproxesul* or oxaceprol* or oxametacin* or oxaprazine* or oxaprozin* or oxicam derivative* or oxindanac* or oxyphenbutazone*).ti,ab. | 16052 |
| 16 | (palifermin* or parcetasal* or parecoxib* or pelubiprofen* or pemedolac* or perisoxal* or phenylbutazone* or phenylbutazone megallate* or picolamine salicylate* or piketoprofen* or pimeprofen* or pipebuzone* or piproxen* or pirazolac* or pirfenidone* or piroxicam* or piroxicam beta cyclodextrin* or pirprofen* or plonmarlimab* or plozalizumab* or polmacoxib* or pralnacasan* or pranoprofen* or prinomide* or prinomide triethanolamine* or proglumetacin* or proquazone* or pyrazinobutazone* or quellor* or rapamycin* or rasagiline* or ravagalimab* or relfovetmab* or reltecimod* or remestemcel L* or resatorvid* or rimacalib* or rimazolium* or risankizumab* or robenacoxib* or rofecoxib* or romazarit* or rosiptor* or rosmarinic acid* or rovazolac* or rozibafusp alfa* or ruxolitinib* or salazosulfapyridine* or salicylic acid* or salnacedin* or salsalate* or satralizumab* or scalaradial* or semapimod* or semorinemab* or sibofimloc* or simufilam* or sudoxicam* or sulfosalicylate samarium* or sulindac* or suprofen* or suxibuzone*).ti,ab. | 6447 |
| 17 | (talniflumate* or tapinarof* or tazofelone* or telazorlimab* or tenidap* or tenosal* or tenosiprol* or tenoxicam* or tepoxalin* or teriflunomide* or tesnatilimab* or tiaprofenic acid* or tiaramide* or tilmacoxib* or tilnoprofen arbamel* or tilomisole* or timegadine* or tioxamast* or tioxaprofen* or tirnovetmab* or tolebrutinib* or tolfenamic acid* or tolmetin* or tomaralimab* or tomicorat* or torudokimab* or tozorakimab* or tralokinumab* or tribuzone* or triethanolamine salicylate* or tropesin* or tryptamide* or ufenamate* or valategrast* or valdecoxib* or valerylsalicylic acid* or vasoactive intestinal polypeptide* or vedaprofen* or velsecorat* or vemircopan* or verramed* or vilobelimab* or vixarelimab* or ximoprofen* or zabedosertib* or zaloglanstat* or zaltoprofen* or zaurategrast* or zidometacin* or zinc salicylate* or zoliprofen* or zomepirac*).ti,ab. | 1562 |
| 18 | exp Colchicine/ | 375 |
| 19 | (Colbenemid* or Colchichin* or colchicum* or colchily* or colchineos* or colchimedio* or colchiquim* or colchisol* or colchysat* or colcin* or colcrys* or colctab* or colgout* or colrefuz* or colsaloid* or Condylon* or gloperba* or goutichine* or goutnil* or kolkicin* or kolkisin* or mitigare* or tolchicine*).ti,ab. | 15 |
| 20 | Prednisone/ | 4110 |
| 21 | (adasone* or acsis* or ancortone* apo-prednisone* or bicortone* or biocortone* or cartancyl* or colisone* or Cortan* or cortancyl* or cortidelt* or cortiprex* or cotone* or cutason* or dacorten* or dacortin* or decortancyl* or decortin* or de-cortisyl* or decortisyl* or dihydrocortisone* or dihydrocortisone* or dekortin or dellacort* or deltacorten* or delta-cortelan* or delta-cortisone* or deltacortisone* or deltacortone* or delta-dome* or delta-prenovis* or deltasone* or delitisone* or deltison* or deltra* or diadreson* or di-adreson* or drazone* or econosone* or encorton* or encortone* or enkorton* or enkortolon* or fernisone* or fiasone* or hostacortin* or insone* or incocortyl* or juvason* or kortancyl* or liquid pred* or lodotra* or lodtra* or lisacort* or me-korti* or meprison* or metacortandracin* or Meticorten* or meticortine* or nisona* or nizon* or novoprednisone* or nurison* or Orasone* or orisane* or panafcort* or paracort* or panasol* or parmenison* or pehacort* or predeltin* or precort* or precortal* or prednicen* or prednicorm* or prednicort* or prednicot* or predni tablinen* or prednidib* or prednilonga* or predniment* or prednison* or prednitone* or prednizon* or prednovister* or presone* or pronison* or pronizon* or pulmison* or rayos* or rectodelt* or rectrocortine* or servisone* or sone$2 or steerometz* or sterapred* or supercortil* or ultracorten* or urtilone* or winpred* or wojtab* or zenadrid*).ti,ab. | 8451 |
| 22 | Methotrexate/ | 4281 |
| 23 | (abitextrate* or abitrexate* or amethopterin* or amethopterine* or ametopterine* or antifolan* or biotrexate* or brimexate* or canceren* or emtexate* or emthexat* or emthexate* or emtrexate* or enthexate* or farmitrexat* or farmotrex* or fauldexato* or folex* or ifamet* or imeth$2 or intradose MTX or jylamvo* or lantarel* or ledertrexate* or lumexon* or maxtrex* or medsatrexate* or metatrexan* or metex* or methoblastin* or methohexate* or methotrate* or methotrexat* or methylaminopterin* or metical* or metoject* or metotressato* or metothrexate* or metotrexat* or metotrexin* or metrex* or metrotex* or mexate* or neotrexate* or nordimet* or novatrex* or otrexup* or rasuvo* or reditrex* or reumatrex* or rheumatrex* or texate* or texorate* or tremetex* or trexall* or trexeron* or trixilem* or xaken* or xatmep* or zexate*).ti,ab. | 11086 |
| 24 | Interleukin-1beta/ | 466 |
| 25 | (ACZ-885* or ACZ885* or Canakinumab* or ilaris*).ti,ab. | 364 |
| 26 | Antibodies, Monoclonal, Humanized/ | 4397 |
| 27 | (pexelizumab or "h5G1.1-SC*").ti,ab. | 53 |
| 28 | Interleukin 1 Receptor Antagonist Protein/ | 331 |
| 29 | (Anakinra* or Antril* or Kineret* or il-1ra* or (interleukin 1 receptor adj1 (antagonist or block* or inhibit*))).ti,ab. | 1153 |
| 30 | (agi-1067* or agi1067* or agz-1067* or agz1067* or probucol succinate* or Succinobucol*).ti,ab. | 13 |
| 31 | (ftx-1821* or ftx1821* or gsk-856553* or gsk856553* or gw-856553* or gw856553* or Losmapimod* or sb-856553* or sb856553*).ti,ab. | 74 |
| 32 | (lc1004* or lc-1004* or inclacumab* or ro4905417* or ro-4905417*).ti,ab. | 20 |
| 33 | (Atreleuton* or a85761* or a-85761* or abt761* or abt-761* or via-2291* or via2291*).ti,ab. | 18 |
| 34 | (darapladib* or sb-480848* or sb480848*).ti,ab. | 74 |
| 35 | (Varespladib* or Varepladib* or ly315920* or ly-315920* or s-5920* or s5920*).ti,ab. | 22 |
| 36 | or/10-35 | 82339 |
| 37 | 9 and 36 | 3464 |

# S4 - Supplementary Table 4. ClinicalTrials.Gov Search

| **Platform: ClinicalTrials.Gov (**[**https://clinicaltrials.gov/**](https://clinicaltrials.gov/)**)**  **Date Searched: March 11, 2022** | |
| --- | --- |
| **Search** | **Results** |
| ( atherosclerosis OR arteriosclerosis OR coronary OR endarterectomy OR thrombectomy OR atherectomy OR embolectomy )  \| ( NSAID OR anti-inflammatory OR colchicine OR prednisone OR methotrexate OR canakinumab OR pexelizumab OR anakinra OR succinobucol OR losmapimod OR inclacumab OR atreleuton OR darapladib OR varespladib ) | 811 |

# S5 - Supplementary Table 5. World Health Organization International Clinical Trials Registry Platform (ICTRP) Search

| **Platform:** **World Health Organization International Clinical Trials Registry Platform (ICTRP) (https://www.who.int/clinical-trials-registry-platform)**  **Date Searched: March 11, 2022** | |
| --- | --- |
| **Search** | **Results** |
| (atherosclerosis OR arteriosclerosis OR coronary OR endarterectomy OR thrombectomy OR atherectomy OR embolectomy) AND (NSAID OR anti-inflammatory OR colchicine OR prednisone OR methotrexate OR canakinumab OR pexelizumab OR anakinra OR succinobucol OR losmapimod OR inclacumab OR atreleuton OR darapladib OR varespladib) | 174 records for 92 trials found! |

# S6 - Supplementary Table 6. Europe PMC Search (<https://europepmc.org/>)

| **Platform:** **Europe PMC (https://europepmc.org/)**  **Date Searched:** **March 11, 2022** | |
| --- | --- |
| **Search** | **Results** |
| TITLE:(athero* OR arteri* OR coronary* OR endarterec* OR thrombect* OR atherect* OR embolect*) OR ABSTRACT:(athero* OR arteri* OR coronary* OR endarterec* OR thrombect* OR atherect* OR embolect*)) AND (TITLE:(NSAID* OR anti-inflamm* OR colchicine* OR prednisone* OR methotrexate* OR canakinumab* OR pexelizumab* OR anakinra* OR succinobucol* OR losmapimod* OR inclacumab* OR atreleuton* OR darapladib* OR varespladib*) OR ABSTRACT:(NSAID* OR anti-inflamm* OR colchicine* OR prednisone* OR methotrexate* OR canakinumab* OR pexelizumab* OR anakinra* OR succinobucol* OR losmapimod* OR inclacumab* OR atreleuton* OR darapladib* OR varespladib*)) AND (PUB_TYPE:"Preprint") | 25 results |

# S7 - Supplementary Table 7. List of Conferences Hand-searched

| **Platforms: Various conference websites**  **Date searched: March 18, 2022** | |
| --- | --- |
| **Conferences of Interest** | **Indexation Status in Embase** |
| European Society of Cardiology (ESC) Congress | Indexed up to and including 2021 |
| American College of Cardiology | Indexed up to and including 2021 |
| American Heart Association | Indexed up to and including 2021 |
| Canadian Cardiovascular Congress | Indexed up to and including 2020    2021 conference website searched for the following terms:  NSAID (1 result)  anti-inflammatory (0 results)  colchicine (0 results)  prednisone (0 results)  methotrexate (0 results)  canakinumab (0 results)  pexelizumab (0 results)  anakinra (0 results)  succinobucol (0 results)  losmapimod (0 results)  inclacumab (0 results)  atreleuton (0 results)  darapladib (0 results)  varespladib (0 results) |

S8 – Supplementary Table 8. Study Characteristics

| Study | Anti-inflammatory agent | Study population | Total number of participants (control) | Lost to follow up (n) | Mean (SD) or median age intervention | Mean (SD) or median age control | Female intervention (fraction) | Female control (fraction) | Length of follow up | Smoking (fraction) intervention | Smoking (fraction) control | HTN (fraction) intervention | HTN (fraction) control | DLD (fraction) intervention | DLD (fraction) control | History of prior ACS/MI (fraction) intervention | History of prior ACS/MI (fraction) control |
| --- | --- | --- | --- | --- | --- | --- | --- | --- | --- | --- | --- | --- | --- | --- | --- | --- | --- |
| **ACS Network** | | | | | | | | | | | | | | | | | |
| Raju 2012 | Colchicine | ACS or ischemic stroke | 80 (40) | 2 | 57.2 (11.7) | 57.2 (8.7) | 6/40 | 3/40 | 1 mo | 31/40 | 32/40 | 19/40 | 15/40 | 19/40 | 19/40 | 8/40 | 6/40 |
| Kleveland 2016 | Tocilizumab | NSTEMI | 121 (59) | 4 | 59.8 (7.7) | 60.1 (9.9) | 9/58 | 5/59 | 6 mo | 15/58 | 17/59 | 26/58 | 17/59 | N/A | N/A | 9/58 | 7/59 |
| Tardif 2019 | Colchicine | MI | 4745 (2379) | 89 | 60.6 (10.7) | 60.5 (10.6) | 472/2366 | 437/2379 | Median 22.6 mo | 708/2366 | 708/2377 | 1185/2366 | 1236/2379 | N/A | N/A | 370/2366 | 397/2379 |
| Broch 2021 | Tocilizumab | STEMI | 199 (98) | 9 | 62 (10) | 60 (9) | 21/101 | 11/98 | 6 mo | 63/101 | 62/98 | 33/101 | 30/98 | 19/101 | 9/98 | N/A | N/A |
| Morton 2014 | Anakinra | NSTE-ACS | 182 (89) | 16 | 61.4 (11.7) | 61.3 (12.3) | 30/93 | 22/89 | 1 yr | 68/93 | 58/89 | 31/93 | 29/89 | 27/93 | 28/89 | 23/93 | 24/89 |
| O'Donoghue 2016 | Losmapimod | NSTEMI, STEMI | 3503 (1758) | 14 | 500/1731 | 532/1758 | 66.5 (9.7) | 66.7 (10) | 3 mo | 464/1731 | 449/1758 | 1268/1731 | 1276/1758 | 985/1731 | 936/1758 | 425/1731 | 426/1758 |
| Nicholls 2014 | Varespladib | ACS | 5145 (2573) | 58 | 61 (10) | 60.7 (9.8) | 691/2572 | 660/2573 | 6 mo | 854/2572 | 860/2573 | 1911/2572 | 1977/2573 | 1255/2572 | 1292/2573 | 769/2572 | 743/2573 |
| Shah 2015 | Colchicine | ACS | 714 (348) | 0 | 66.1 (9.7) | 65.8 (10.7) | 30/366 | 24/348 | 1 mo | N/A | N/A | N/A | N/A | N/A | N/A | N/A | N/A |
| Mewton 2021 | Colchicine | STEMI | 192 (91) | 31 | 59.0 (10.6) | 60.9 (10.4) | 21/101 | 17/91 | 3 mo | 44/101 | 39/91 | 30/101 | 29/91 | 29/101 | 34/91 | N/A | N/A |
| Rusnak 2001 | LeukArrest (Hu23F2G) | STEMI | 88 (19) | 28 | 59 (12.6) | 62.4 (13.5) | 7/21 | 5/19 | 4-6 wk | 12/21 | 13/19 | 10/21 | 5/19 | 10/21 | 11/19 | 1/21 | 3/19 |
| O'Donoghue 2014 | Darapladib | NSTEMI, STEMI | 6522 |  | 64 | 64 | 1657/6504 | 1669/6522 | 3 yr | 1227/6504 | 1245/6522 | 4793/6504 | 4762/6522 | 4191/6504 | 4165/6522 | 2013/6504 | 2033/6522 |
| Talasaz 2009 | Colchicine | STEMI | 102 (101) | 0 | N/A | N/A | N/A | N/A | 1 mo | N/A | N/A | N/A | N/A | N/A | N/A | N/A | N/A |
| Newby 2014 (low dose) | Losmapimod 7.5 | NSTEMI | 334 (135) | 9 | 62 | 64 | 52/199 | 45/135 | 3 mo | 64/199 | 46/135 | 145/199 | 97/135 | 118/199 | 74/135 | 35/199 | 33/135 |
| Newby 2014 (low dose) | Losmapimod 15 | NSTEMI | 327 (195) | 9 | 63 | 64 | 57/192 | 45/135 | 3 mo | 56/192 | 46/135 | 136/192 | 97/135 | 115/192 | 74/135 | 46/192 | 33/135 |
| Rosenson 2010 | Varespladib | ACS | 625 (313) | 1 | 67 (11) | 64 (12) | 12/44 | 11/46 | 6 mo | N/A | N/A | N/A | N/A | N/A | N/A | N/A | N/A |
| Akodad 2017 | Colchicine | STEMI | 23 (21) | N/A | N/A | N/A | N/A | N/A | 1 mo | N/A | N/A | N/A | N/A | N/A | N/A | N/A | N/A |
| Akrami 2021 | Colchicine | STEMI | 120 (129) | 3 | 56.9 (7.6) | 56.9 (7.5) | 34/120 | 42/129 | 6 mo | 52/120 | 49/129 | 52/120 | 59/129 | 37/120 | 36/129 | 14/120 | 15/129 |
| Altman 2002 | COX-2 inhibitor | NSTE-ACS | 60 (60) | 11 | 61 | 60.7 | 29/60 | 23/60 | 3 mo | 24/60 | 30/60 | 22/60 | 27/60 | 17/60 | 17/60 | 11/60 | 11/60 |
| Abbate 2020 | Anakinra | STEMI | 35 | 0 | 53 | 55 | 9/33 | 5/31 | 1 yr | N/A | N/A | N/A | N/A | N/A | N/A | N/A | N/A |
| Jolly 2024 | Colchicine | ACS | 3528 (3534) | 43 | 60.6 (10.3) | 60.7 (10.3) | 725/3528 | 713/3534 | 3 yr | 1461/3528 | 1423/3534 | 1620/3528 | 1630/3534 | N/A | N/A | 309/3528 | 324/3534 |
| Bouleti 2024 | Colchicine | ACS | 101 (91) | 6 | 59 (10.6) | 60.9 (10.4) | 21/101 | 17/91 | 1 yr | 44/101 | 39/91 | 30/101 | 29/91 | 29/101 | 34/91 | N/A | N/A |
| Hosseini 2022 | Colchcine | STEMI | 161 (160) | 39 | 58.7 (10.4) | 59.0 (11.2) | 34/161 | 33/160 | 1 yr | 65/161 | 72/160 | 65/161 | 62/160 | 29/161 | 38/160 | N/A | N/A |
| **CAD Network** | | | | | | | | | | | | | | | | | |
| Rosenson 2011 | Varespladib | CAD | 135 (46) | 11 | 67 (9) | 64 (12) | 14/45 | 11/46 | 8 wk | N/A | N/A | N/A | N/A | N/A | N/A | N/A | N/A |
| Ribichini 2013 | Prednisone | Consecutive patients undergoing coronary angiography | 375 (125) | 10 | 63.3 (9.03) | 64.1 (9.7) | 17/125 | 23/125 | 4 yr | 79/125 | 85/125 | 91/125 | 84/125 | 71/125 | 79/125 | 28/125 | 18/125 |
| Kang 2012 | Celecoxib | Angina pectoris or positive stress test with native coronary artery lesions for which DES feasible | 909 (454) | 119 | 63.2 (9) | 63.3 (9.1) | 142/449 | 155/454 | 3 mo | 113/449 | 111/454 | 305/449 | 298/454 | 193/449 | 198/454 | 20/449 | 18/454 |
| Stahl 2016 | Inclacumab | Undergoing CABG | 384 (144) | N/A | 62.1 (9.2) | 62.8 (8.2) | 16/148 | 15/144 | 1 yr | N/A | N/A | N/A | N/A | N/A | N/A | N/A | N/A |
| Choudhury 2016 | Canakinumab | Atherosclerotic disease and either T2DM or IGT | 189 (94) | 3 | 61.7 (7.8) | 61.9 (6.9) | 13/95 | 14/94 | 1 yr | 7/95 | 7/94 | 81/95 | 83/94 | N/A | N/A | N/A | N/A |
| Shah 2020 | Colchicine | Suspected ACS referred for possible PCI | 400 (194) | 0 | 65.9 (9.9) | 66.6 (10.2) | 13/206 | 13/194 | 1 mo | 148/206 | 134/194 | 192/206 | 175/194 | 182/206 | 173/194 | 51/206 | 52/194 |
| Nidorf 2020 | Colchicine | CAD | 5065 (2760) | 0 | 65.8 (8.4) | 65.9 (8.7) | 457/2762 | 389/2760 | 28.6 mo | 318/2762 | 330/2760 | 1421/2762 | 1387/2760 | 2670/2762 | 2665/2760 | 2323/2762 | 2335/2760 |
| Stone 1989 | Methylpred and prednisone | Restenosis following prior PTCA | 102 (50) | N/A | N/A | N/A | N/A | N/A | 7 d | N/A | N/A | N/A | N/A | N/A | N/A | N/A | N/A |
| Ridker 2017 | Canakinumab 50 | Previous MI, CRP >2 | 5514 (3344) | 27 | 61.1 (10.1) | 61.1 (10.0) | 541/2170 | 865/3344 | 48 mo | 531/2170 | 765/3344 | 1751/2170 | 2644/3344 | 2038/2169 | 3132/3344 | 2170/2170 | 3344/3344 |
| 2017 | Canakinumab 150 | Previous MI, CRP >2 | 5823 (3344) | 27 | 61.2 (10.0) | 61.1 (10.0) | 575/2284 | 865/3344 | 48 mo | 534/2284 | 765/3344 | 1814/2284 | 2644/3344 | 2114/2280 | 3132/3344 | 2284/2284 | 3344/3344 |
| 2017 | Canakinumab 300 | Previous MI, CRP >2 | 5607 (3344) | 27 | 61.1 (10.1) | 61.1 (10.0) | 606/2263 | 865/3344 | 48 mo | 536/2263 | 765/3344 | 1799/2263 | 2644/3344 | 2113/2259 | 3132/3344 | 2263/2263 | 3344/3344 |
| van Groenendael 2021 | EA-230 | Elective CABG | 180 (89) | 1 | 67 (0.9) | 68 (1) | 10/90 | 12/89 | N/A | N/A | N/A | N/A | N/A | N/A | N/A | N/A | N/A |
| Stability 2014 | Darapladib | Previous MI, PCI, CABG, multivessel CAD | 15828 (7904) | 149 | 65 | 65 | 1461/7924 | 1506/7904 | 3.7 yrs | 1572/7924 | 1656/7904 | N/A | N/A | N/A | N/A | 4681/7924 | 4642/7904 |
| Ridker 2019 | Methotrexate | CAD | 4786 (2395) | N/A | 65.6 | 66 | 461/2391 | 437/2395 | Median 2.3 yrs | 267/2391 | 270/2395 | 2153/2391 | 2169/2395 | N/A | N/A | N/A | N/A |
| Ott 2003 | Parecoxib/valdecoxib | Undergoing CABG | 462 (151) | 118 | 60.3 (8.2) | 61.3 (8) | 46/311 | 16/151 | Mean 12.5 d | 168/311 | 85/151 | 38/311 | 17/151 | N/A | N/A | 128/311 | 66/151 |
| Versaci 2002 | Steroid | Stenting with CRP >0.5 mg/dl post-procedure | 83 (42) | N/A | 63 (9) | 65 (9) | 9/41 | 5/42 | 1 yr | 12/41 | 11/42 | 16/41 | 15/42 | 18/41 | 18/42 | N/A | N/A |
| Shernan 2004 | Pexelizumab | Referred for PCI | 914 (306) | 0 | N/A | N/A | N/A | N/A | 1 mo | N/A | N/A | N/A | N/A | N/A | N/A | N/A | N/A |
| Nidorf 2013 | Colchicine | CAD | 532 (250) | 0 | 66 (9.6) | 67 (9.2) | 31/282 | 228/250 | 3 yr | 10/282 | 14/250 | N/A | N/A | N/A | N/A | 64/282 | 61/250 |
| Tardif 2008 | Succinobucol | PCI | 465 (111) | 26 | 61 (9) | 61 (9) | 71/354 | 21/111 | 12 mo | 64/354 | 18/111 | 211/354 | 70/111 | 345/354 | 104/111 | 99/354 | 39/111 |
| Serruys 2008 | Darapladib | CAD | 330 (151) | 4 | 59.4 (9.8) | 57.3 (10.9) | 42/170 | 25/151 | 1 yr | 64/172 | 57/151 | 115/172 | 89/151 | 108/172 | 95/151 | 51/172 | 49/151 |
| Chung 2010 | Celecoxib | Angina pectoris or positive stress test, and native coronary lesions for which DES implantation feasible | 189 (137) | 4 | 62.6 (9.1) | 64.2 (9.2) | 42/130 | 47/137 | 6 mo | 23/130 | 27/137 | 90/130 | 91/137 | 88/130 | 103/137 | 5/130 | 8/137 |
| Dzavik 2010 | Varespladib | Elective PCI | 72 (72) | 8 | 62.8 (9.9) | 64.0 (9.9) | 14/72 | 11/72 | 1 mo | N/A | N/A | 54/72 | 55/72 | N/A | N/A | 21/72 | 20/72 |

ACS=acute coronary syndrome, CABG=coronary artery bypass graft, CAD=coronary artery disease, CRP=C-reactive protein, DLD=dyslipidemia, HTN=hypertension, IGT=impaired glucose tolerance, MI=myocardial infarction, NSTE-ACS=non-ST-elevation myocardial infarction, NSTEMI=non-ST-elevation myocardial infarction, PCI=primary coronary intervention, SD=standard deviation, STEMI=ST-elevation myocardial infarction, T2DM=type 2 diabetes mellitus

S9 - Supplementary Table 9. ACS – Summary of Intervention Characteristics

| **Treatment** | **# Studies** | **# Events** | **# Patients** | **Aggregate Rate** | **Min. Rate** | **Max. Rate** |
| --- | --- | --- | --- | --- | --- | --- |
| **Control** | 21 | 1477 | 14614 | 0.1011 | 0.0096 | 0.3714 |
| **Anakinra** | 2 | 27 | 157 | 0.1720 | 0.1398 | 0.2188 |
| **Colchicine** | 7 | 189 | 2,947 | 0.0641 | 0.0278 | 0.1485 |
| **Tocilizumab** | 2 | 12 | 159 | 0.0755 | 0.0517 | 0.0891 |
| **Losmapimod low dose** | 2 | 168 | 1930 | 0.0870 | 0.0803 | 0.1457 |
| **Losmapimod high dose** | 1 | 35 | 192 | 0.1823 | 0.1823 | 0.1823 |
| **LeukArrest low dose** | 1 | 2 | 21 | 0.0952 | 0.0952 | 0.0952 |
| **LeukArrest high dose** | 1 | 1 | 20 | 0.0500 | 0.0500 | 0.0500 |
| **Varespladib** | 2 | 164 | 2883 | 0.0569 | 0.0544 | 0.0772 |
| **Darapladib** | 1 | 903 | 6504 | 0.1388 | 0.1388 | 0.1388 |
| **NSAIDs** | 1 | 8 | 60 | 0.1333 | 0.1333 | 0.1333 |

S10 - Supplementary Table 10. ACS – Summary of Direct Comparison Characteristics

| **Comparison** | **# Studies** | **# Patients** | **# Events** |
| --- | --- | --- | --- |
| **Control vs. Colchicine** | 7 | 5,900 | 441 |
| **Control vs. Tocilizumab** | 2 | 316 | 28 |
| **Control vs. Anakinra** | 2 | 281 | 44 |
| **Control vs. Losmapimod low dose** | 2 | 3,823 | 316 |
| **Control vs. Varespladib** | 2 | 5,769 | 277 |
| **Control vs. LeukArrest low dose** | 1 | 40 | 3 |
| **Control vs. LeukArrest high dose** | 1 | 39 | 2 |
| **LeukArrest low dose vs. LeukArrest high dose** | 1 | 41 | 3 |
| **Control vs. Darapladib** | 1 | 13,026 | 1,813 |
| **Control vs. Losmapimod high dose** | 1 | 327 | 60 |
| **Losmapimod low dose vs. Losmapimod high dose** | 1 | 391 | 64 |
| **Control vs. NSAIDs** | 1 | 120 | 28 |

S11 - Supplementary Table 11. Stable CAD – Summary of Intervention Characteristics

| **Treatment** | **# Studies** | **# Events** | **# Patients** | **Aggregate Rate** | **Min. Rate** | **Max. Rate** |
| --- | --- | --- | --- | --- | --- | --- |
| **Control** | 20 | 2059.5 | 18819 | 0.1094 | 0.0109 | 0.3571 |
| **Varespladib** | 2 | 9 | 121 | 0.0744 | 0.0204 | 0.1111 |
| **Steroid** | 3 | 27 | 218 | 0.1239 | 0.0732 | 0.1600 |
| **NSAIDs** | 3 | 64 | 883 | 0.0725 | 0.0675 | 0.0769 |
| **Inclacumab** | 1 | 21 | 148 | 0.1419 | 0.1419 | 0.1419 |
| **Canakinumab low** | 1 | 313 | 2170 | 0.1442 | 0.1442 | 0.1442 |
| **Canakinumab medium** | 2 | 330 | 2379 | 0.1387 | 0.1053 | 0.1401 |
| **Canakinumab high** | 1 | 322 | 2263 | 0.1423 | 0.1423 | 0.1423 |
| **Colchicine** | 3 | 226 | 3250 | 0.0695 | 0.0532 | 0.1165 |
| **EA-230** | 1 | 11 | 91 | 0.1209 | 0.1209 | 0.1209 |
| **Darapladib** | 2 | 798 | 8096 | 0.0986 | 0.0970 | 0.1686 |
| **Methotrexate** | 1 | 170 | 2391 | 0.0711 | 0.0711 | 0.0711 |
| **Pexelizumab bolus** | 1 | 15 | 308 | 0.0487 | 0.0487 | 0.0487 |
| **Pexelizumab bolus and infusion** | 1 | 10 | 300 | 0.0333 | 0.0333 | 0.0333 |
| **Succinobucol** | 1 | 22 | 354 | 0.0621 | 0.0621 | 0.0621 |

S12 - Supplementary Table 12. Stable CAD – Summary of Direct Comparison Characteristics

| **Comparison** | **# Studies** | **# Patients** | **# Events** |
| --- | --- | --- | --- |
| **Control vs. Varespladib** | 2 | 239 | 13 |
| **Control vs. Steroid** | 3 | 435 | 79 |
| **Control vs. NSAIDs** | 3 | 1,625 | 134 |
| **Control vs. Inclacumab** | 1 | 292 | 41 |
| **Control vs. Canakinumab medium** | 2 | 5,817 | 872 |
| **Control vs. Colchicine** | 3 | 6,454 | 555 |
| **Control vs. Canakinumab low** | 1 | 5,514 | 848 |
| **Control vs. Canakinumab high** | 1 | 5,607 | 857 |
| **Canakinumab low vs. Canakinumab medium** | 1 | 4,454 | 633 |
| **Canakinumab low vs. Canakinumab high** | 1 | 4,433 | 635 |
| **Canakinumab medium vs. Canakinumab high** | 1 | 4,547 | 642 |
| **Control vs. EA-230** | 1 | 180 | 26 |
| **Control vs. Darapladib** | 2 | 16,151 | 1,641 |
| **Control vs. Methotrexate** | 1 | 4,786 | 337 |
| **Control vs. Pexelizumab bolus** | 1 | 614 | 22 |
| **Control vs. Pexelizumab bolus and infusion** | 1 | 606 | 17 |
| **Pexelizumab bolus vs. Pexelizumab bolus and infusion** | 1 | 608 | 25 |
| **Control vs. Succinobucol** | 1 | 465 | 33 |

# S13 - Supplementary Table 13. Confidence in the results.

S14 - Supplementary Table 14. ACS – MACE Evidence Summary Table

| Treatment | Pair-wise Meta-Analysis, OR (95% CI) versus placebo | NMA, Odds Ratio (95% CrI) versus placebo | Probability Best (%) for Outcome | Rank for Outcome | SUCRA |
| --- | --- | --- | --- | --- | --- |
| Colchicine | 0.72 (0.45, 1.15) | 0.77 (0.62-0.95) | 0.8% | 3.3 | 0.7850 |
| NSAIDs | 0.31 (0.12, 0.77) | 0.30 (0.11, 0.43) | 66.4% | 1.5 | 0.9546 |
| Anakinra | 3.45 (1.08, 11.03) | 3.70 (1.26, 13.78) | 0% | 6.9 | 0.0718 |
| Tocilizumab | 0.72 (0.33, 1.59) | 0.72 (0.32, 1.56) | 4.5% | 4.0 | 0.7189 |
| Losmapimod low dose | 1.01 (0.68, 1.51) | 1.09 (0.86, 1.37) | 0% | 7.0 | 0.4428 |
| Losmapimod high dose | 0.98 (0.56, 1.73) | 1.21 (0.75, 1.97) | 0% | 7.8 | 0.3748 |
| LeukArrest low dose | 1.89 (0.16, 22.75) | 2.22 (0.16, 77.17) | 4.3% | 8.4 | 0.2916 |
| LeukArrest high dose | 0.95 (0.06, 16.31) | 0.92 (0.02, 36.30) | 23.95% | 5.8 | 0.3748 |
| Varespladib | 1.29 (1.00, 1.66) | 1.49 (1.16, 1.91) | 0% | 9.5 | 0.2261 |
| Darapladib | 0.99 (0.90, 1.10) | 0.99 (0.90, 1.10) | 0% | 5.9 | 0.5501 |

CI=confidence interval, CrI=credible interval, OR=odds ratio, SUCRA=surface under the cumulative ranking

# S15 - Supplementary Table 15. Chronic CAD – MACE Evidence Summary Table

| Treatment | Pair-wise Meta-Analysis, OR (95% CI) versus placebo | NMA, Odds Ratio (95% CrI) versus placebo | Probability Best (%) for Outcome | Rank for Outcome | SUCRA |
| --- | --- | --- | --- | --- | --- |
| Colchicine | 0.58 (0.35, 0.99) | 0.65 (0.54, 0.77) | 1.4% | 3.5 | 0.8370 |
| NSAIDs | 0.85 (0.31, 2.33) | 0.81 (0.56, 1.16) | 0.5% | 6.4 | 0.6292 |
| Steroids | 0.47 (0.15, 1.50) | 0.44 (0.26, 0.72) | 51.1% | 1.8 | 0.9577 |
| Succinobucol | 0.60 (0.28, 1.28) | 0.61 (0.29, 1.31) | 15.6% | 4.4 | 0.7843 |
| EA-230 | 0.68 (0.29, 1.57) | 0.67 (0.28, 1.54) | 12.7% | 5.3 | 0.7147 |
| Canakinumab medium-dose | 0.88 (0.70, 1.10) | 0.87 (0.75, 1.00) | 0% | 7.1 | 0.5857 |
| Canakinumab high-dose | 0.87 (0.75, 1.01) | 0.87 (0.75, 1.01) | 0% | 7.4 | 0.5656 |
| Canakinumab low-dose | 0.88 (0.76, 1.03) | 0.89 (0.76, 1.03) | 0% | 7.6 | 0.5360 |
| Darapladib | 0.93 (0.84, 1.03) | 0.93 (0.84, 1.03) | 0% | 8.9 | 0.4560 |
| Inclacumab | 1.03 (0.53, 1.98) | 1.03 (0.53, 2.04) | 0.7% | 9.6 | 0.3985 |
| Methotrexate | 1.02 (0.82, 1.27) | 1.02 (0.82, 1.28) | 0% | 10.6 | 0.3306 |
| Pexelizumab bolus and infusion | 1.47 (0.55, 3.92) | 1.49 (0.55, 4.17) | 0.8% | 11.8 | 0.2372 |
| Pexelizumab bolus | 2.19 (0.88, 5.44) | 2.22 (0.92, 5.88) | 0% | 14.0 | 0.0788 |
| Varespladib | 2.88 (0.11, 72.41) | 2.94 (0.82, 12.5) | 17.3% | 10.8 | 0.0723 |

CI=confidence interval, CrI=credible interval, OR=odds ratio, SUCRA=surface under the cumulative ranking

# S16 - Supplementary Table 16. ACS – MACE Evidence Summary Table for Studies with ≥30 Days of Follow Up and ≥30 Days of Treatment

| Treatment | Pair-wise Meta-Analysis, OR (95% CI) versus placebo | NMA, Odds Ratio (95% CrI) versus placebo | Probability Best (%) for Outcome | Rank for Outcome | SUCRA |
| --- | --- | --- | --- | --- | --- |
| NSAIDs |  | 0.28 (0.10 – 0.70) |  |  | 0.9959 |
| Colchicine |  | 0.80 (0.61 –1.05) |  |  | 0.8239 |
| Darapladib |  | 0.91 (0.71 – 1.18) |  |  | 0.5920 |
| Losmapimod low dose |  | 1.09 (0.86 – 1.37) |  |  | 0.4518 |
| Losmapimod high dose |  | 1.22 (0.74 – 1.96) |  |  | 0.3676 |
| Varespladib |  | 1.49 (1.16 – 1.89) |  |  | 0.1720 |
| Anakinra |  | 3.57 (1.16 – 14.3) |  |  | 0.0234 |

CI=confidence interval, CrI=credible interval, OR=odds ratio, SUCRA=surface under the cumulative ranking

# S17 - Supplementary Table 17. ACS – MACE Evidence Summary Table for Studies Published 2010 or Later with ≥30 Days of Follow Up and ≥30 Days of Treatment

| Treatment | Pair-wise Meta-Analysis, OR (95% CI) versus placebo | NMA, Odds Ratio (95% CrI) versus placebo | Probability Best (%) for Outcome | Rank for Outcome | SUCRA |
| --- | --- | --- | --- | --- | --- |
| Colchicine |  | 0.87  (0.77 – 0.99) |  |  | 0.9594 |
| Darapladib |  | 0.99  (0.90 – 1.10) |  |  | 0.6911 |
| Losmapimod low dose |  | 1.09  (0.86 – 1.37) |  |  | 0.5227 |
| Losmapimod high dose |  | 1.20  (0.74 – 1.96) |  |  | 0.4309 |
| Varespladib |  | 1.47  (1.16 – 1.89) |  |  | 0.2009 |
| Anakinra |  | 3.57 (1.18 – 14.3) |  |  | 0.0267 |

CI=confidence interval, CrI=credible interval, OR=odds ratio, SUCRA=surface under the cumulative ranking

# S18 - Supplementary Table 18. Chronic CAD – MACE Evidence Summary Table for Studies with ≥30 Days of Follow Up and ≥30 Days of Treatment

| Treatment | Pair-wise Meta-Analysis, OR (95% CI) versus placebo | NMA, Odds Ratio (95% CrI) versus placebo | Probability Best (%) for Outcome | Rank for Outcome | SUCRA |
| --- | --- | --- | --- | --- | --- |
| Steroid |  | 0.37  (0.21 – 0.65) |  |  | 0.9616 |
| Colchicine |  | 0.63  (0.52 – 0.76) |  |  | 0.7922 |
| NSAIDs |  | 0.64  (0.43 – 0.96) |  |  | 0.7573 |
| Succinobucol |  | 0.61  (0.29 – 1.36) |  |  | 0.7202 |
| Canakinumab medium |  | 0.87  (0.75 – 1.01) |  |  | 0.5152 |
| Canakinumab high |  | 0.88  (0.75 – 1.02) |  |  | 0.4962 |
| Canakinumab low |  | 0.89  (0.76 – 1.04) |  |  | 0.4662 |
| Inclacumab |  | 1.02  (0.53 – 2.02) |  |  | 0.3195 |
| Varespladib |  | 2.61  (0.05 – 723.10) |  |  | 0.2755 |
| Methotrexate |  | 1.02  (0.81 – 1.27) |  |  | 0.2418 |
| Darapladib |  | 1.02  (0.92 – 1.14) |  |  | 0.2095 |

CI=confidence interval, CrI=credible interval, OR=odds ratio, SUCRA=surface under the cumulative ranking

# S19 - Supplementary Table 19. Chronic CAD – MACE Evidence Summary Table for Studies Published 2010 or Later with ≥30 Days of Follow Up and ≥30 Days of Treatment

| Treatment | Pair-wise Meta-Analysis, OR (95% CI) versus placebo | NMA, Odds Ratio (95% CrI) versus placebo | Probability Best (%) for Outcome | Rank for Outcome | SUCRA |
| --- | --- | --- | --- | --- | --- |
| Steroid |  | 0.37  (0.21 – 0.65) |  |  | 0.9616 |
| Colchicine |  | 0.63  (0.52 – 0.76) |  |  | 0.7922 |
| NSAIDs |  | 0.64  (0.43 – 0.96) |  |  | 0.7573 |
| Succinobucol |  | 0.61  (0.29 – 1.36) |  |  | 0.7202 |
| Canakinumab medium |  | 0.87  (0.75 – 1.01) |  |  | 0.5152 |
| Canakinumab high |  | 0.88  (0.75 – 1.02) |  |  | 0.4962 |
| Canakinumab low |  | 0.89  (0.76 – 1.04) |  |  | 0.4662 |
| Inclacumab |  | 1.02  (0.53 – 2.02) |  |  | 0.3195 |
| Varespladib |  | 2.61  (0.05 – 723.10) |  |  | 0.2418 |
| Methotrexate |  | 1.02  (0.81 – 1.27) |  |  | 0.2095 |

CI=confidence interval, CrI=credible interval, OR=odds ratio, SUCRA=surface under the cumulative ranking

# S20 - Supplementary Figure 1. Inconsistency plot for stable CAD network. Shows posterior mean deviance of the individual data points from fitted consistency and inconsistency models.


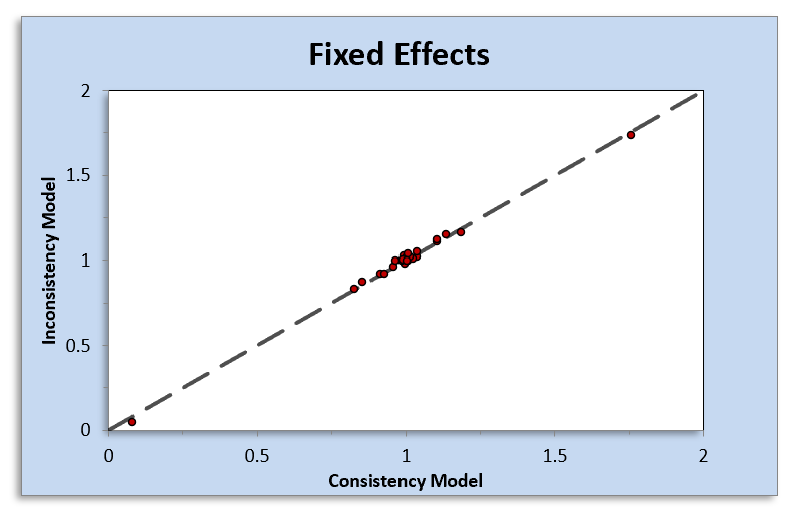


# S21 - Supplementary Figure 2. Inconsistency plot for ACS network. Shows posterior mean deviance of the individual data points from fitted consistency and inconsistency models.

# **Secondary Outcomes**

*Secondary Outcome: Individual subcomponents of MACE*

**ACS Network**

We evaluated each subcomponent of MACE in a network meta-analysis. The ACS network looking at MI included 23 RCTs^1-23^, representing 13 interventions in addition to placebo/control with 41,608 patients in the analysis. The evidence network for MI is similar, albeit larger than for MACE, and included 8 studies (Rosenson 2011^1^, Moreira 2017^2^, Tardif 2013^4^, Brochier 1993^9^, Tardif 2008^11^, Versaci 2002^13^, Armstrong 2007^22^, Ulander 2021^20^) that were not in the original network for MACE. Tocilizumab and NSAIDs were associated with a reduced risk of MI relative to placebo/control.

The ACS network looking at non-fatal stroke included 15 RCTs^3,4,6-8,10-12,14,16,21,22,24-26^, representing 8 interventions in addition to placebo/control. A total of 41,796 patients were included in the analysis. The evidence network for non-fatal stroke included 6 studies (Tardif 2013^4^, Tong 2021^24^, Tardif 2008^11^, Granger 2003^25^, Mahaffey 2003^26^, Armstrong 2007^22^) that were not in the original network for MACE. Only colchicine-use was associated with a reduced risk of non-fatal stroke relative to placebo/control.

The acute CAD network looking at cardiovascular death included 12 RCTs^5,6,8,16,18-20,24,27^, representing 9 interventions in addition to placebo/control. A total of 34,624 patients were included in the analysis. The evidence network for cardiovascular death was again similar but smaller than for MACE, and included 4 studies (Nakamura 2009^27^, Tong 2021^24^, Tardif 2008^11^, Ulander 2021^20^) that were not in the original network for MACE. No interventions were associated with a reduced risk of cardiac death relative to placebo/control.

**Stable CAD Network**

The stable CAD network looking at MI included 20 RCTs^1,28-46^, representing 12 interventions in addition to placebo/control. The evidence network for MI is similar, albeit larger than for MACE, and included 5 studies (Wong 2006^30^, Koo 2007^38^, Smith 2011^39^, Brown 2004^43^, Dzavik 2010^46^) that were not in the original network for MACE. Only colchicine and the 150mg-dose of canakinumab were associated with a reduced risk of MI relative to placebo/control.

The stable CAD network looking at non-fatal stroke included 11 RCTs^33,34,36,37,41,42,44,47-50^, representing 8 interventions in addition to placebo/control. The evidence network for non-fatal stroke included 4 studies (Hauser 2016^47^, Shah 2020^33^, Verrier 2004^41^, Cheruku 2004^49^) that were not in the original network for MACE. No interventions were associated with a reduced risk of non-fatal stroke relative to placebo/control.

The stable CAD network looking at cardiovascular death included 10 RCTs^29,34,36-38,40,44,45,48,51^, representing 8 interventions in addition to placebo/control. A total of 33,841 patients were included in the analysis. The evidence network for cardiovascular death was again similar but smaller than for MACE and included only one study (Koo 2007^38^) that was not in the original network for MACE. Only colchicine was associated with a reduced risk of cardiac death relative to placebo/control.

*Secondary Outcomes: Adverse therapy effects*

**ACS Network**

The ACS network looking at clinically significant infection included 11 RCTs^2,4-8,12,16,52-54^ (references), representing 10 interventions in addition to placebo/control. The evidence network for clinically significant infection is similar, albeit smaller than for MACE (with 15,890 patients), and included 4 studies (Moreira 2017^2^, Tardif 2013^4^, Rusnak 2001^53^) that were not in the original network for MACE. None of the included interventions were associated with an increased risk of infection relative to placebo/control.

The ACS network looking at malignancy included 4 RCTs^6,9,12,14^, representing 3 interventions (colchicine, NSAIDs, darapladib) in addition to placebo/control. The evidence network for malignancy is significantly smaller than for MACE and included one study (Brochier 1993^9^) that was not in the original network for MACE. None of the included interventions were associated with an increased risk of malignancy relative to placebo/control.

The ACS network looking at diarrhea and GI upset included 10 RCTs^2,3,6,8,9,12,14,33,37,55^, representing 5 interventions (colchicine, NSAIDs, darapladib, methotrexate, losmapimod) in addition to placebo/control. The evidence network for diarrhea/GI upset is significantly smaller than for MACE, and included four studes (Moreira 2017^2^, O’Keefe 1992^55^, Brochier 1993^9^, Stability 2014^37^) that was not in the original network for MACE. None of the included interventions were associated with an increased risk of diarrhea/GI upset relative to placebo/control.

**Stable CAD Network**

The Stable CAD network looking at clinically significant infection included 10 RCTs^30,32,34,36,40-42,47,48,56^, representing 8 interventions in addition to placebo/control. The evidence network for clinically significant infection is similar, albeit smaller than for MACE, and included 4 studies (Wong 2006^30^, Zarpelon 2016^56^, Hauser 2016^47^, Verrier 2005^41^) that were not in the original network for MACE. None of the included interventions were associated with an increased risk of infection relative to placebo/control.

The stable CAD network looking at malignancy included 3 RCTs^34,36,48^, representing 5 interventions (colchicine, canakinumab low dose, canakinumab medium dose, canakinumab high-dose, and methotrexate) in addition to placebo/control. The evidence network for malignancy is significantly smaller than for MACE. Methotrexate use was associated with an increased risk of malignancy relative to placebo/control.

The stable CAD network looking at diarrhea and GI upset included 11 RCTs^1,32-34,36,37,41,42,47,57,58^, representing 10 interventions (colchicine, varespladib low dose, varespladib high dose, canakinumab medium-dose, salsalate, darapladib low dose, darapladib medium dose, darapladib high dose, pexelizumab, and methotrexate) in addition to placebo/control. The evidence network for malignancy is similar but smaller than for MACE. Darapladib low and high dose use was associated with a reduced risk of GI upset/diarrhea relative to placebo/control. Methotrexate and colchicine-use were associated with an increased risk of diarrhea/GI upset compared with placebo/control.

**References**

1 Rosenson, R. S., Elliott, M., Stasiv, Y., Hislop, C. & Investigators, P. I. Randomized trial of an inhibitor of secretory phospholipase A2 on atherogenic lipoprotein subclasses in statin-treated patients with coronary heart disease. *Eur Heart J* **32**, 999-1005, doi:10.1093/eurheartj/ehq374 (2011).

2 Moreira, D. M., Lueneberg, M. E., da Silva, R. L., Fattah, T. & Gottschall, C. A. M. MethotrexaTE THerapy in ST-Segment Elevation MYocardial InfarctionS: A Randomized Double-Blind, Placebo-Controlled Trial (TETHYS Trial). *J Cardiovasc Pharmacol Ther* **22**, 538-545, doi:10.1177/1074248417699884 (2017).

3 Raju, N. C. *et al.* Effect of colchicine compared with placebo on high sensitivity C-reactive protein in patients with acute coronary syndrome or acute stroke: a pilot randomized controlled trial. *J Thromb Thrombolysis* **33**, 88-94, doi:10.1007/s11239-011-0637-y (2012).

4 Tardif, J. C. *et al.* Effects of the P-selectin antagonist inclacumab on myocardial damage after percutaneous coronary intervention for non-ST-segment elevation myocardial infarction: results of the SELECT-ACS trial. *J Am Coll Cardiol* **61**, 2048-2055, doi:10.1016/j.jacc.2013.03.003 (2013).

5 Kleveland, O. *et al.* Effect of a single dose of the interleukin-6 receptor antagonist tocilizumab on inflammation and troponin T release in patients with non-ST-elevation myocardial infarction: a double-blind, randomized, placebo-controlled phase 2 trial. *Eur Heart J* **37**, 2406-2413, doi:10.1093/eurheartj/ehw171 (2016).

6 Tardif, J. C. *et al.* Efficacy and Safety of Low-Dose Colchicine after Myocardial Infarction. *N Engl J Med* **381**, 2497-2505, doi:10.1056/NEJMoa1912388 (2019).

7 Broch, K. *et al.* Randomized Trial of Interleukin-6 Receptor Inhibition in Patients With Acute ST-Segment Elevation Myocardial Infarction. *J Am Coll Cardiol* **77**, 1845-1855, doi:10.1016/j.jacc.2021.02.049 (2021).

8 O'Donoghue, M. L. *et al.* Effect of Losmapimod on Cardiovascular Outcomes in Patients Hospitalized With Acute Myocardial Infarction: A Randomized Clinical Trial. *JAMA* **315**, 1591-1599, doi:10.1001/jama.2016.3609 (2016).

9 Brochier, M. L. Evaluation of flurbiprofen for prevention of reinfarction and reocclusion after successful thrombolysis or angioplasty in acute myocardial infarction. The Flurbiprofen French Trial. *Eur Heart J* **14**, 951-957, doi:10.1093/eurheartj/14.7.951 (1993).

10 Nicholls, S. J. *et al.* Varespladib and cardiovascular events in patients with an acute coronary syndrome: the VISTA-16 randomized clinical trial. *JAMA* **311**, 252-262, doi:10.1001/jama.2013.282836 (2014).

11 Tardif, J. C. *et al.* Effects of the antioxidant succinobucol (AGI-1067) on human atherosclerosis in a randomized clinical trial. *Atherosclerosis* **197**, 480-486, doi:10.1016/j.atherosclerosis.2006.11.039 (2008).

12 Mewton, N. *et al.* Effect of Colchicine on Myocardial Injury in Acute Myocardial Infarction. *Circulation* **144**, 859-869, doi:10.1161/CIRCULATIONAHA.121.056177 (2021).

13 Versaci, F. *et al.* Immunosuppressive Therapy for the Prevention of Restenosis after Coronary Artery Stent Implantation (IMPRESS Study). *J Am Coll Cardiol* **40**, 1935-1942, doi:10.1016/s0735-1097(02)02562-7 (2002).

14 O'Donoghue, M. L. *et al.* Effect of darapladib on major coronary events after an acute coronary syndrome: the SOLID-TIMI 52 randomized clinical trial. *JAMA* **312**, 1006-1015, doi:10.1001/jama.2014.11061 (2014).

15 Monakier, D. *et al.* Rofecoxib, a COX-2 inhibitor, lowers C-reactive protein and interleukin-6 levels in patients with acute coronary syndromes. *Chest* **125**, 1610-1615, doi:10.1378/chest.125.5.1610 (2004).

16 Rosenson, R. S. *et al.* Effects of varespladib methyl on biomarkers and major cardiovascular events in acute coronary syndrome patients. *J Am Coll Cardiol* **56**, 1079-1088, doi:10.1016/j.jacc.2010.06.015 (2010).

17 Akodad, M. *et al.* COLIN trial: Value of colchicine in the treatment of patients with acute myocardial infarction and inflammatory response. *Arch Cardiovasc Dis* **110**, 395-402, doi:10.1016/j.acvd.2016.10.004 (2017).

18 Akrami, M. *et al.* Effects of colchicine on major adverse cardiac events in next 6-month period after acute coronary syndrome occurrence; a randomized placebo-control trial. *BMC Cardiovasc Disord* **21**, 583, doi:10.1186/s12872-021-02393-9 (2021).

19 Altman, R. *et al.* Efficacy assessment of meloxicam, a preferential cyclooxygenase-2 inhibitor, in acute coronary syndromes without ST-segment elevation: the Nonsteroidal Anti-Inflammatory Drugs in Unstable Angina Treatment-2 (NUT-2) pilot study. *Circulation* **106**, 191-195, doi:10.1161/01.cir.0000021599.56755.a1 (2002).

20 Ulander, L. *et al.* Hydroxychloroquine reduces interleukin-6 levels after myocardial infarction: The randomized, double-blind, placebo-controlled OXI pilot trial. *Int J Cardiol* **337**, 21-27, doi:10.1016/j.ijcard.2021.04.062 (2021).

21 Abbate, A. *et al.* Interleukin-1 Blockade Inhibits the Acute Inflammatory Response in Patients With ST-Segment-Elevation Myocardial Infarction. *J Am Heart Assoc* **9**, e014941, doi:10.1161/JAHA.119.014941 (2020).

22 Investigators, A. A. *et al.* Pexelizumab for acute ST-elevation myocardial infarction in patients undergoing primary percutaneous coronary intervention: a randomized controlled trial. *JAMA* **297**, 43-51, doi:10.1001/jama.297.1.43 (2007).

23 Talasaz AH, J. Y., Hosseini SH. P4611 Colchicine before percutaneous coronary intervention in acute myocardial infarction. *Eur Heart J* **40**, 2822, doi:10.1093/eurheartj/ehz745.0994 (2019).

24 Tong, D. C. *et al.* Colchicine in Patients With Acute Coronary Syndrome: Two-Year Follow-Up of the Australian COPS Randomized Clinical Trial. *Circulation* **144**, 1584-1586, doi:10.1161/CIRCULATIONAHA.121.054610 (2021).

25 Granger, C. B. *et al.* Pexelizumab, an anti-C5 complement antibody, as adjunctive therapy to primary percutaneous coronary intervention in acute myocardial infarction: the COMplement inhibition in Myocardial infarction treated with Angioplasty (COMMA) trial. *Circulation* **108**, 1184-1190, doi:10.1161/01.CIR.0000087447.12918.85 (2003).

26 Mahaffey, K. W. *et al.* Effect of pexelizumab, an anti-C5 complement antibody, as adjunctive therapy to fibrinolysis in acute myocardial infarction: the COMPlement inhibition in myocardial infarction treated with thromboLYtics (COMPLY) trial. *Circulation* **108**, 1176-1183, doi:10.1161/01.CIR.0000087404.53661.F8 (2003).

27 Nakamura, Y. *et al.* Effect of edaravone on plasma monocyte chemoattractant protein-1 levels in patients with acute myocardial infarction. *J Cardiol* **54**, 416-424, doi:10.1016/j.jjcc.2009.07.001 (2009).

28 Ribichini, F. *et al.* Long-term clinical follow-up of the multicentre, randomized study to test immunosuppressive therapy with oral prednisone for the prevention of restenosis after percutaneous coronary interventions: Cortisone plus BMS or DES veRsus BMS alone to EliminAte Restenosis (CEREA-DES). *Eur Heart J* **34**, 1740-1748, doi:10.1093/eurheartj/eht079 (2013).

29 Kang, H. J. *et al.* Effects of celecoxib on restenosis after coronary intervention and evolution of atherosclerosis (Mini-COREA) trial: celecoxib, a double-edged sword for patients with angina. *Eur Heart J* **33**, 2653-2661, doi:10.1093/eurheartj/ehs001 (2012).

30 Wong, P. S., Asmat, A., Chan, Y. H. & Lee, C. N. A randomized, double-blind, placebo-controlled trial of a COX-2 inhibitor (Rofecoxib) in patients undergoing coronary artery bypass surgery. *Interact Cardiovasc Thorac Surg* **5**, 101-104, doi:10.1510/icvts.2005.118455 (2006).

31 Stahli, B. E. *et al.* Effects of P-Selectin Antagonist Inclacumab in Patients Undergoing Coronary Artery Bypass Graft Surgery: SELECT-CABG Trial. *J Am Coll Cardiol* **67**, 344-346, doi:10.1016/j.jacc.2015.10.071 (2016).

32 Choudhury, R. P. *et al.* Arterial Effects of Canakinumab in Patients With Atherosclerosis and Type 2 Diabetes or Glucose Intolerance. *J Am Coll Cardiol* **68**, 1769-1780, doi:10.1016/j.jacc.2016.07.768 (2016).

33 Shah, B. *et al.* Effects of Acute Colchicine Administration Prior to Percutaneous Coronary Intervention: COLCHICINE-PCI Randomized Trial. *Circ Cardiovasc Interv* **13**, e008717, doi:10.1161/CIRCINTERVENTIONS.119.008717 (2020).

34 Nidorf, S. M. *et al.* Colchicine in Patients with Chronic Coronary Disease. *N Engl J Med* **383**, 1838-1847, doi:10.1056/NEJMoa2021372 (2020).

35 Stone, G. W. *et al.* A randomized trial of corticosteroids for the prevention of restenosis in 102 patients undergoing repeat coronary angioplasty. *Cathet Cardiovasc Diagn* **18**, 227-231, doi:10.1002/ccd.1810180407 (1989).

36 Ridker, P. M. *et al.* Low-Dose Methotrexate for the Prevention of Atherosclerotic Events. *N Engl J Med* **380**, 752-762, doi:10.1056/NEJMoa1809798 (2019).

37 Investigators, S. *et al.* Darapladib for preventing ischemic events in stable coronary heart disease. *N Engl J Med* **370**, 1702-1711, doi:10.1056/NEJMoa1315878 (2014).

38 Koo, B. K. *et al.* Effect of celecoxib on restenosis after coronary angioplasty with a Taxus stent (COREA-TAXUS trial): an open-label randomised controlled study. *Lancet* **370**, 567-574, doi:10.1016/S0140-6736(07)61295-1 (2007).

39 Smith, P. K. *et al.* Effects of C5 complement inhibitor pexelizumab on outcome in high-risk coronary artery bypass grafting: combined results from the PRIMO-CABG I and II trials. *J Thorac Cardiovasc Surg* **142**, 89-98, doi:10.1016/j.jtcvs.2010.08.035 (2011).

40 Ott, E. *et al.* Efficacy and safety of the cyclooxygenase 2 inhibitors parecoxib and valdecoxib in patients undergoing coronary artery bypass surgery. *J Thorac Cardiovasc Surg* **125**, 1481-1492, doi:10.1016/s0022-5223(03)00125-9 (2003).

41 Verrier, E. D. *et al.* Terminal complement blockade with pexelizumab during coronary artery bypass graft surgery requiring cardiopulmonary bypass: a randomized trial. *JAMA* **291**, 2319-2327, doi:10.1001/jama.291.19.2319 (2004).

42 Shernan, S. K. *et al.* Impact of pexelizumab, an anti-C5 complement antibody, on total mortality and adverse cardiovascular outcomes in cardiac surgical patients undergoing cardiopulmonary bypass. *Ann Thorac Surg* **77**, 942-949; discussion 949-950, doi:10.1016/j.athoracsur.2003.08.054 (2004).

43 Brown, D. L. *et al.* Clinical and biochemical results of the metalloproteinase inhibition with subantimicrobial doses of doxycycline to prevent acute coronary syndromes (MIDAS) pilot trial. *Arterioscler Thromb Vasc Biol* **24**, 733-738, doi:10.1161/01.ATV.0000121571.78696.dc (2004).

44 Nidorf, S. M., Eikelboom, J. W., Budgeon, C. A. & Thompson, P. L. Low-dose colchicine for secondary prevention of cardiovascular disease. *J Am Coll Cardiol* **61**, 404-410, doi:10.1016/j.jacc.2012.10.027 (2013).

45 Chung, J. W. *et al.* Long-term outcome of adjunctive celecoxib treatment after paclitaxel-eluting stent implantation for the complex coronary lesions: two-year clinical follow-up of COREA-TAXUS trial. *Circ Cardiovasc Interv* **3**, 243-248, doi:10.1161/CIRCINTERVENTIONS.109.889881 (2010).

46 Dzavik, V. *et al.* The sPLA2 Inhibition to Decrease Enzyme Release after Percutaneous Coronary Intervention (SPIDER-PCI) trial. *Circulation* **122**, 2411-2418, doi:10.1161/CIRCULATIONAHA.110.950733 (2010).

47 Hauser, T. H. *et al.* Effect of Targeting Inflammation With Salsalate: The TINSAL-CVD Randomized Clinical Trial on Progression of Coronary Plaque in Overweight and Obese Patients Using Statins. *JAMA Cardiol* **1**, 413-423, doi:10.1001/jamacardio.2016.0605 (2016).

48 Ridker, P. M. *et al.* Antiinflammatory Therapy with Canakinumab for Atherosclerotic Disease. *N Engl J Med* **377**, 1119-1131, doi:10.1056/NEJMoa1707914 (2017).

49 Cheruku, K. K. *et al.* Efficacy of nonsteroidal anti-inflammatory medications for prevention of atrial fibrillation following coronary artery bypass graft surgery. *Prev Cardiol* **7**, 13-18, doi:10.1111/j.1520-037x.2004.3117.x (2004).

50 Serruys, P. W. *et al.* Effects of the direct lipoprotein-associated phospholipase A(2) inhibitor darapladib on human coronary atherosclerotic plaque. *Circulation* **118**, 1172-1182, doi:10.1161/CIRCULATIONAHA.108.771899 (2008).

51 Ribichini, F. *et al.* Effects of prednisone on biomarkers of tubular damage induced by radiocontrast in interventional cardiology. *J Nephrol* **26**, 586-593, doi:10.5301/jn.5000266 (2013).

52 Morton, A. C. *et al.* The effect of interleukin-1 receptor antagonist therapy on markers of inflammation in non-ST elevation acute coronary syndromes: the MRC-ILA Heart Study. *Eur Heart J* **36**, 377-384, doi:10.1093/eurheartj/ehu272 (2015).

53 Rusnak, J. M. *et al.* An anti-CD11/CD18 monoclonal antibody in patients with acute myocardial infarction having percutaneous transluminal coronary angioplasty (the FESTIVAL study). *Am J Cardiol* **88**, 482-487, doi:10.1016/s0002-9149(01)01723-4 (2001).

54 Armstrong, P. W. & Granger, C. B. Pexelizumab and the APEX AMI trial. *JAMA* **297**, 1881; author reply 1881-1882, doi:10.1001/jama.297.17.1881-b (2007).

55 O'Keefe, J. H., Jr. *et al.* Ineffectiveness of colchicine for the prevention of restenosis after coronary angioplasty. *J Am Coll Cardiol* **19**, 1597-1600, doi:10.1016/0735-1097(92)90624-v (1992).

56 Zarpelon, C. S. *et al.* Colchicine to Reduce Atrial Fibrillation in the Postoperative Period of Myocardial Revascularization. *Arq Bras Cardiol* **107**, 4-9, doi:10.5935/abc.20160082 (2016).

57 Deftereos, S. *et al.* Colchicine treatment for the prevention of bare-metal stent restenosis in diabetic patients. *J Am Coll Cardiol* **61**, 1679-1685, doi:10.1016/j.jacc.2013.01.055 (2013).

58 Mohler, E. R., 3rd *et al.* The effect of darapladib on plasma lipoprotein-associated phospholipase A2 activity and cardiovascular biomarkers in patients with stable coronary heart disease or coronary heart disease risk equivalent: the results of a multicenter, randomized, double-blind, placebo-controlled study. *J Am Coll Cardiol* **51**, 1632-1641, doi:10.1016/j.jacc.2007.11.079 (2008).
